# Phase‐Resolved Defect Transport Mechanisms Governing Asynchronous Ordering in a Eutectic High‐Entropy Alloy

**DOI:** 10.1002/advs.75539

**Published:** 2026-05-07

**Authors:** Huiwen Yao, Qingshuang Ma, Jie Xiong, Jing Bai, Huijun Li, Qiuzhi Gao

**Affiliations:** ^1^ School of Materials Science and Engineering Northeastern University Shenyang P. R. China; ^2^ School of Resources and Materials Northeastern University at Qinhuangdao Qinhuangdao P. R. China; ^3^ Materials Genome Institute Shanghai University Shanghai P. R. China; ^4^ Faculty of Engineering and Information Sciences University of Wollongong Wollongong New South Wales Australia

**Keywords:** asynchronous ordering, eutectic high‐entropy alloys, phase‐resolved diffusion, point defect, vacancy‐interstitial cooperation

## Abstract

Eutectic high‐entropy alloys (EHEAs) exhibit asynchronous phase evolution under identical thermal conditions, yet the underlying mechanisms remain unclear. Here, we investigated the isothermal annealing of AlCoCrCuFeNi alloy using phase‐resolved experiments and atomistic simulations. Electron microscopy revealed sharply contrasting structural pathways: the initially disordered BCC‐FeCr phase progressively developed long‐range order, whereas the B2‐NiAl phase retained its macroscopically ordered framework while undergoing local structural relaxation. Molecular dynamics and Monte Carlo simulations traced this discrepancy to phase‐dependent defect energetics. In B2‐NiAl, low vacancy formation and migration energies enable efficient vacancy‐mediated transport, promoting local structural refinement without disrupting the ordered lattice. In BCC‐FeCr, constrained vacancy transport together with accessible low‐energy interstitial configurations gives rise to a cooperative vacancy‐interstitial transport mechanism required for the disorder‐to‐order transition. These heterogeneous defect transport mechanisms govern asynchronous ordering in EHEAs and provide a phase‐resolved basis for designing thermally stable complex alloys.

## Graphical abstract

Phase‐resolved experiments and atomistic simulations reveal asynchronous ordering behaviors in a eutectic high‐entropy alloy during isothermal annealing. Distinct defect transport mechanisms are identified in coexisting B2 and BCC phases, showing that vacancy and interstitial mediated diffusion governs phase‐dependent thermal stability.

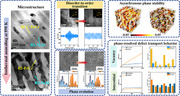

## 1 Introduction

Eutectic high‐entropy alloys (EHEAs) have attracted considerable attention because of their excellent castability and unique multiphase microstructures [1, 2]. EHEAs combine the advantages of eutectic alloys with the multicomponent effects of high‐entropy alloys (HEAs), making them promising for high‐temperature structural applications. Conventional HEAs typically comprise five or more elements in near‐equimolar ratios, often forming single‐phase solid solutions [3]. However, such single‐phase design strategies are increasingly recognized as limited when thermal stability and diffusion‐controlled structural evolution under prolonged high‐temperature exposure are considered [4, 5]. In contrast, EHEAs intentionally adopt multiphase eutectic structures, in which coexisting phases with distinct crystal structures and chemical states are spatially intertwined.

The discovery of EHEAs has increasingly shifted from empirical alloy development toward more systematic and predictive design strategies. In particular, pseudo‐ternary eutectic frameworks, high‐throughput CALPHAD calculations, machine‐learning‐assisted optimization, and broader data‐driven screening approaches have accelerated the identification of promising eutectic compositions from the vast compositional space of complex concentrated alloys [6, 7, 8, 9, 10]. Although these approaches are highly effective in locating candidate alloy systems with desirable phase constitutions and eutectic characteristics, they do not directly resolve the phase‐specific kinetic processes that govern microstructural stability during thermal exposure. A mechanistic understanding of how individual phases evolve under service‐relevant conditions therefore remains essential for translating computationally identified compositions into thermally stable structural materials.

Among EHEAs, Al‐Co‐Cr‐Fe‐Ni‐based systems have been widely reported to form eutectic microstructures involving face‐centered cubic (FCC) and body‐centered cubic (BCC) constituents [11, 12, 13, 14]. Their phase constitution is highly sensitive to composition, especially to Al, which stabilizes BCC and B2 phases [15, 16]. Cu further enhances elemental partitioning and chemical heterogeneity because of its limited solubility in transition metals [17, 18]. Accordingly, AlCoCrCuFeNi is a representative heterogeneous EHEA in which ordered B2 and disordered BCC‐derived constituents coexist [19], making it an ideal model system suitable for investigating phase‐dependent evolution during thermal exposure.

Microstructural stability is critical for EHEAs during thermal exposure. Thermal treatment activates diffusion‐controlled processes such as coarsening, precipitation, ordering, and elemental redistribution [20, 21, 22, 23]. These changes do not necessarily proceed uniformly across coexisting phases, which may evolve at different rates because of differences in crystal structure, chemical partitioning, local ordering state, and defect energetics [1, 24, 25]. Here, asynchronous phase evolution refers to distinct timescales and sequences of structural and ordering changes under identical thermal conditions. Similar non‐uniform evolution has been reported in other eutectic HEAs, particularly in lamellar systems containing FCC/B2 or FCC/BCC constituents, where coexisting phases show different decomposition behavior, selective recrystallization, precipitate evolution, or phase‐stability changes during heating [26, 27, 28]. BCC/B2 systems are especially instructive because disordered BCC and ordered B2 phases provide distinct transport environments under the same annealing conditions.

As the fundamental kinetic mechanism underlying structural evolution, atomic diffusion is particularly complex in multiphase EHEAs [29]. Phase‐specific compositions and structures can produce diffusion behaviors distinct from that in conventional alloys. In particular, disordered BCC‐A2 and ordered B2 phases create distinct transport environments: the latter may favor vacancy‐mediated diffusion, whereas the former can exhibit broader migration‐barrier distributions and more diverse defect‐assisted pathways [30, 31]. Long‐range ordering (LRO) and short‐range ordering (SRO) can further modify atomic migration barriers and diffusion pathways [32, 33]. Previous studies have mainly addressed diffusion in single‐phase HEAs, emphasizing average behavior rather than phase‐specific migration mechanisms in chemically partitioned multiphase systems [34, 35]. Therefore, a phase‐resolved framework that connects defect transport to asynchronous evolution in eutectic alloys remains lacking [36, 37, 38].

In this work, experimental characterization, molecular dynamics (MD), and Monte Carlo (MC) simulations were combined to investigate AlCoCrCuFeNi alloy during isothermal annealing. By directly comparing disordered BCC‐FeCr and ordered B2‐NiAl phases, this study traces the observed asynchronous evolution to their intrinsically different defect energetics and transport responses under the same thermal conditions. This work therefore establishes a phase‐resolved mechanistic framework that directly connects asynchronous ordering behavior in eutectic HEAs with phase‐specific defect energetics and atomic transport pathways.

## 2 Results

### 2.1 Phase Constitution and Microstructural Stability

**FIGURE 1:**
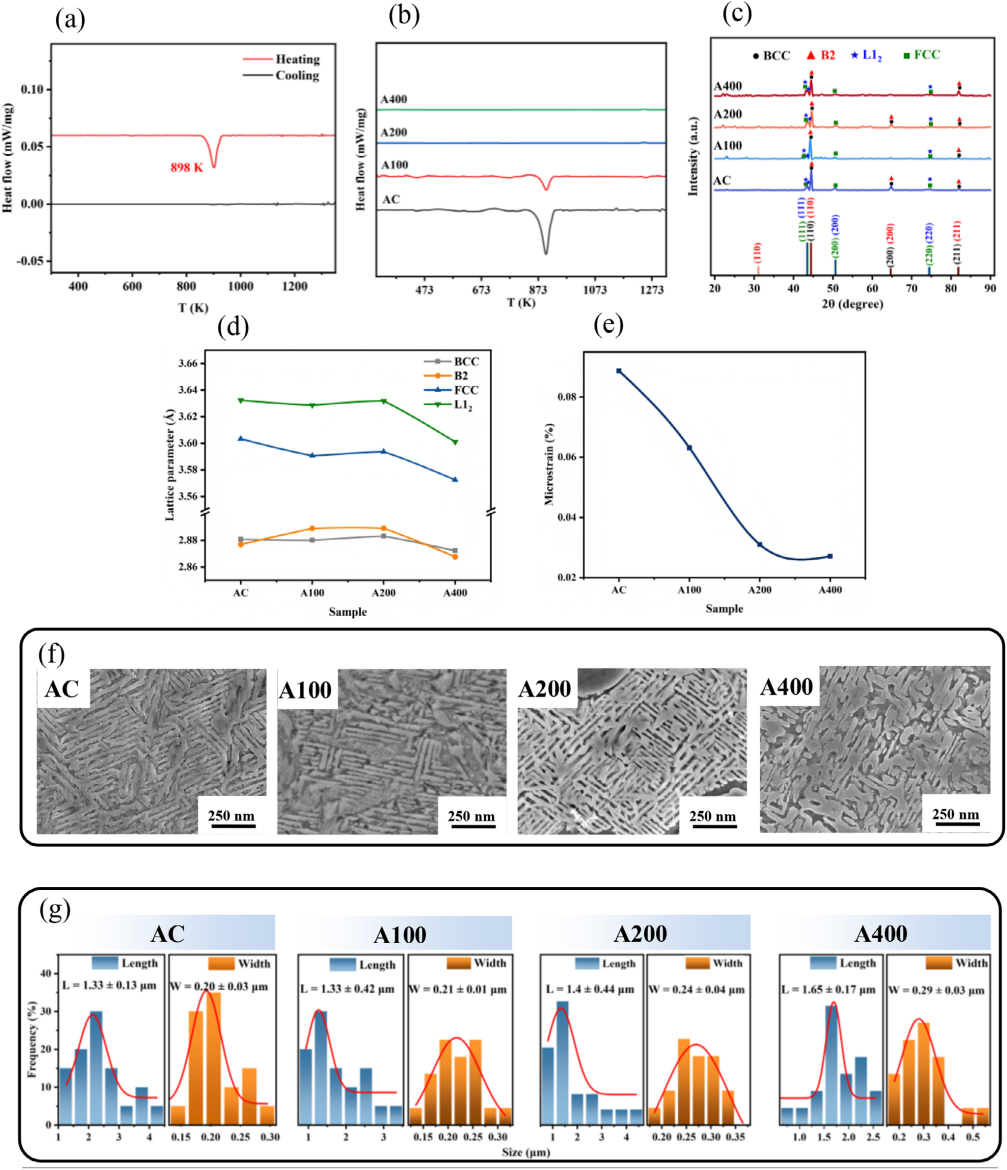
Phase evolution and microstructure of the EHEA after different annealing times, (a) DSC heating and cooling curves of the AC sample, (b) DSC heating curves of EHEA samples after different annealing times, (c) XRD patterns of samples, (d) calculated lattice parameters, (e) microstrain estimated based on XRD patterns, (f) SEM microstructures of the EHEA samples, and (g) phase size of the light‐gray precipitate phase from SEM results.

As shown in Figure 1a, the AC sample exhibited an irreversible endothermic peak at 898 K in the differential scanning calorimetry (DSC) curve. This temperature was selected as the annealing condition to capture the structural evolution associated with this endothermic response. The samples were denoted as as‐cast (AC), 100 h (A100), 200 h (A200), and 400 h (A400) based on the annealing time at 898 K. The peak intensity decreased after 100 h and disappeared in the A200/A400 samples (Figure 1b). No exothermic signals were detected in any annealed sample (Figure S1a). This progressive suppression of the endothermic peak reflects a reduction in structural responsiveness, suggesting that structural adjustments under these conditions were completed within 200 h. Figure 1c shows the X‐ray diffraction (XRD) patterns of the AC and annealed samples. The absence of new major diffraction peaks indicates that the overall multiphase constitution was retained during annealing. At the same time, subtle but systematic evolution of the diffraction profiles is observed (Figure S1b). As shown in Figure 1d,e, refinement and peak‐broadening analysis revealed slight lattice‐parameter adjustments together with a continuous reduction in microstrain estimated by the Williamson–Hall method, indicating progressive structural relaxation during annealing [39]. These diffraction changes provide supporting evidence for structural evolution, while the phase‐specific ordering characteristics and spatial distribution are resolved by subsequent microscopic analysis. The microstructural characteristics of the AC and annealed samples are presented in Figure 1f. A dual‐phase structure, composed of alternating light‐gray and dark regions, was retained in all samples. Modest morphological evolution was observed during annealing, while the phase constitution remained unchanged. Quantitative analysis (Figure 1g) showed that the light‐gray phase widened progressively, leading to a reduced aspect ratio and a more uniform morphology. These results indicate slight coarsening during annealing, while the overall dual‐phase morphology was retained.

### 2.2 Nano‐Scale Structural Evolution

**FIGURE 2:**
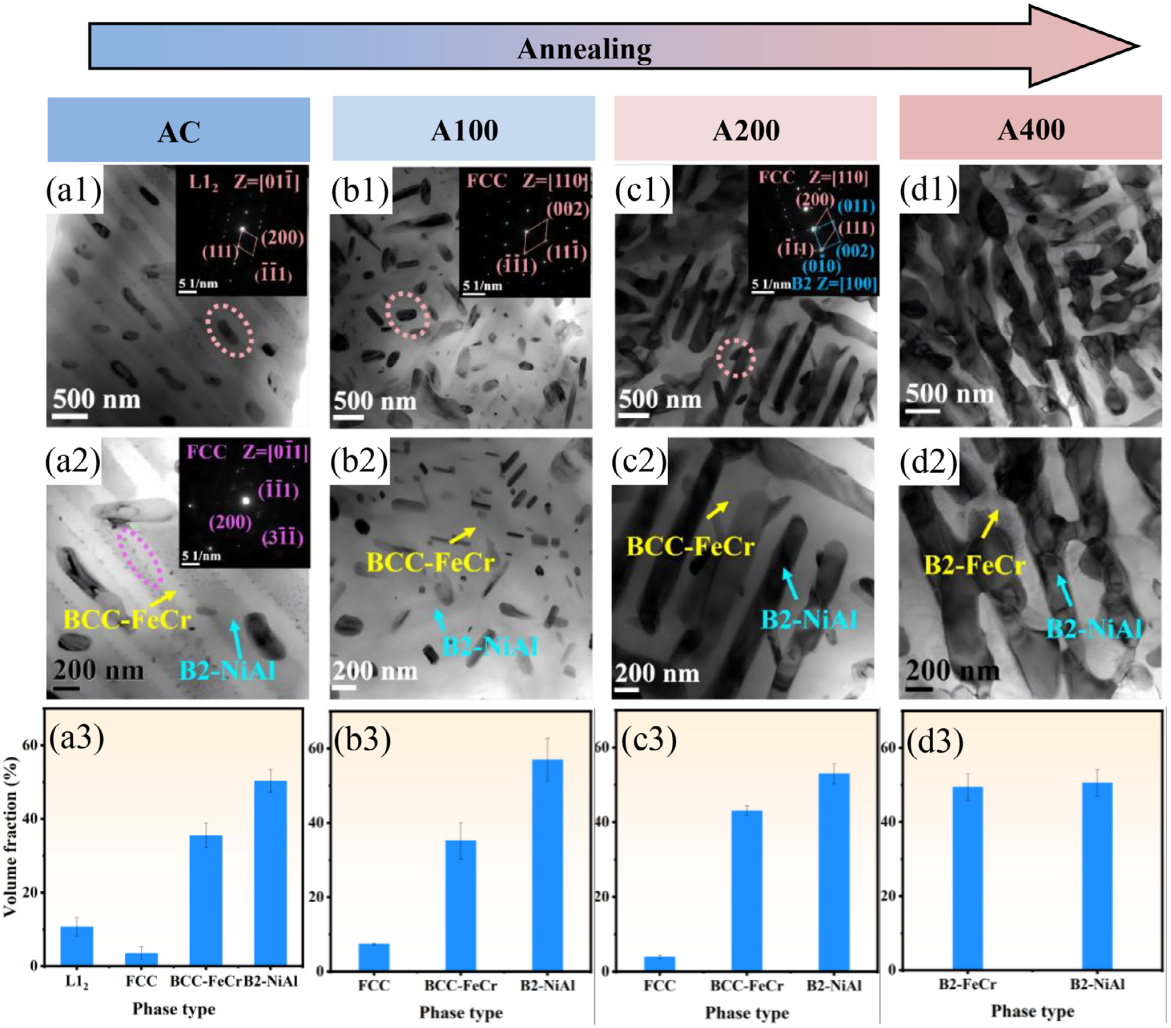
Microstructure and phase composition, bright‐field TEM images, SAED patterns of the EHEA samples and quantitative volume fractions of the constituent phases. (a1–a3) AC sample, (b1–b3) A100 sample, (c1–c3) A200 sample, (d1–d3) A400 sample.

The microstructural evolution and phase compositions of the AC and annealed samples were characterized using bright‐field transmission electron microscopy (TEM), and the corresponding selected area electron diffraction (SAED) patterns are shown in Figure 2a1–d2. The associated phase compositions are listed in Table 1. In the AC sample, the microstructure consisted of a dark B2‐NiAl matrix and a light‐gray disordered BCC‐FeCr phase aligned along the <110> direction. Rod‐shaped Cu‐rich particles were distributed within the B2 matrix (Figure 2a1). The SAED pattern along the [011̄] zone axis showed L1_2_ superlattice reflections, and FCC nanoparticles were observed at the B2/BCC interfaces (Figure 2a2). In the A100 sample, the B2‐NiAl/BCC‐FeCr two‐phase microstructure was preserved (Figure 2b,b2). The dual‐phase B2/BCC framework remained intact. However, the L1_2_‐ordered Cu‐rich phase transformed into an FCC structure, accompanied by the disappearance of interfacial nanoparticles and a reduction in volume fraction (Figure 2b3). In the A200 sample, the Cu‐rich particles further decreased, and a transitional state involving a disordered FCC structure was observed. As quantified in Figure 2c3, the Cu‐rich fraction continuously decreased, while the BCC‐FeCr fraction increased and the B2‐NiAl fraction remained nearly constant. The Cu‐rich phase evolved from L1_2_‐ordered domains in the AC state to FCC and then to transitional structures, accompanied by a continuous decrease in volume fraction, until it could no longer be detected in the A400 sample.

**TABLE 1:** Crystal structure and chemical composition (in at. %) of different phases in AC, A100, A200, and A400 samples (compositional error: ± 3 at. %).

Samples	Phase	Al	Cr	Fe	Ni	Cu	Co
**AC**	BCC‐FeCr	1.29	49.92	29.41	2.45	0.67	16.23
	B2‐NiAl	29.33	2.71	11.91	30.50	6.46	19.05
	L1_2_	4.45	0.3	0.06	3.21	91.91	0.07
	FCC	3.14	0.64	0.10	4.15	89.43	0.16
**A100**	BCC‐FeCr	0.88	53.80	25.33	1.35	0.34	18.30
	B2‐NiAl	30.62	1.94	15.03	26.61	3.54	22.26
	FCC	3.57	0.62	1.86	2.89	89.24	1.82
**A200**	BCC‐FeCr	0.68	51.14	27.00	2.20	0.32	18.67
	B2‐NiAl	32.94	1.44	11.39	29.14	4.17	20.93
	FCC	9.02	0.89	3.52	9.24	72.16	5.16
**A400**	Ordered BCC‑FeCr	0.75	50.01	27.13	2.86	0.68	18.54
	B2‐NiAl	28.08	2.49	12.78	30.61	5.29	20.72

**FIGURE 3:**
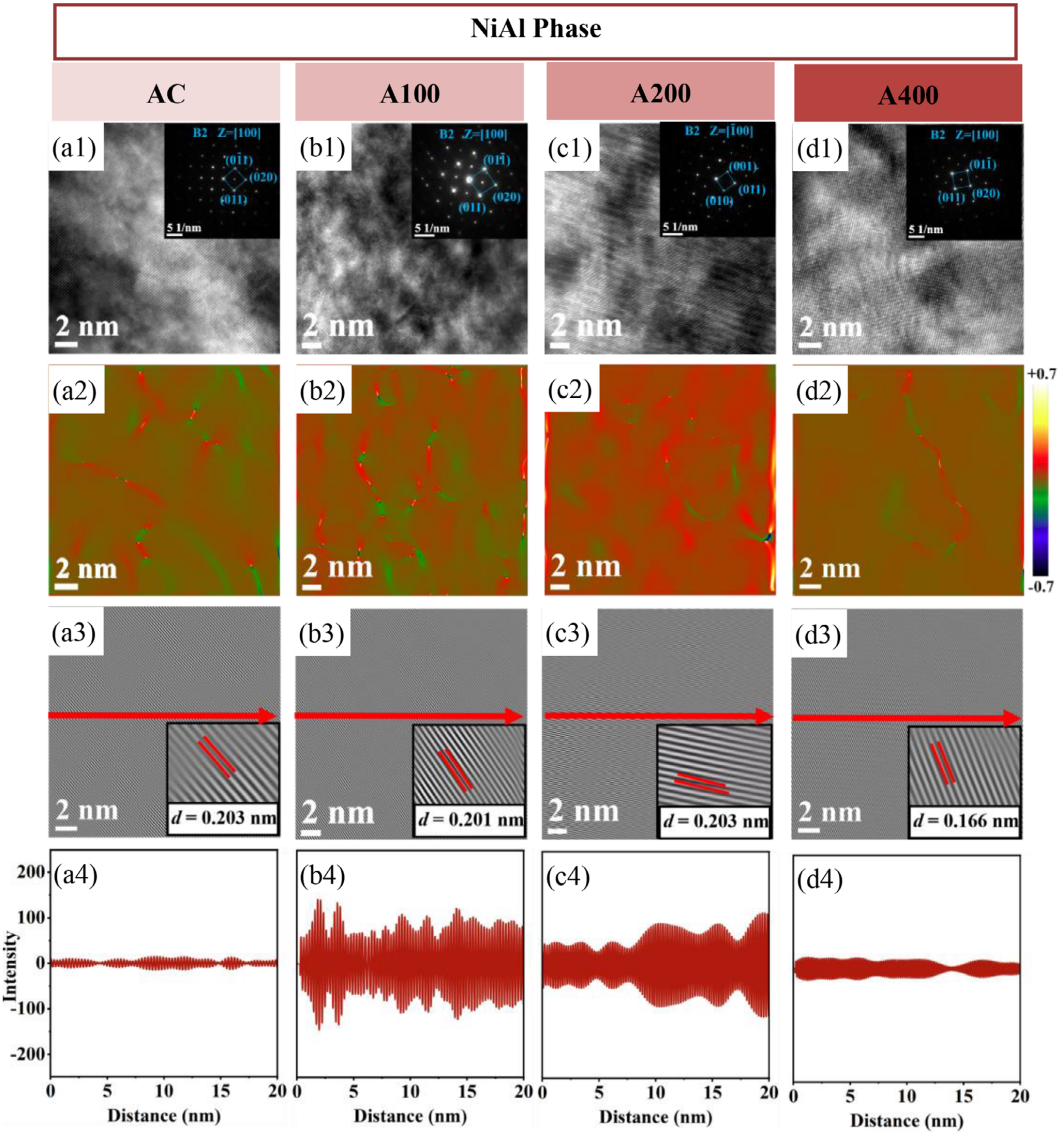
HR‐TEM images with corresponding SAED patterns, strain maps showing horizontal normal strain (*ε_xx_*), iFFT of the HR‐TEM pattern, and iFFT intensity profiles of the NiAl phase in EHEA samples after different annealing times: (a1–a4) AC sample, (b1–b4) A100 sample, (c1–c4) A200 sample, (d1–d4) A400 sample.

The B2 phase exhibited a decoupled structural evolution characterized by the retention of its LRO structure alongside progressive local relaxation. The LRO structure was maintained throughout annealing, as confirmed by invariant SAED patterns with a lattice parameter of 2.85 Å (Figure 3a1–d1). However, local structural adjustments were clearly evident. Qualitatively, high‐resolution transmission electron microscopy (HR‐TEM) lattice fringes became increasingly continuous. This progressive local relaxation was quantitatively corroborated by geometric phase analysis (GPA) and inverse Fourier transform (iFFT) patterns. Specifically, localized high‐contrast strain concentrations (red and blue regions) in the AC and A100 GPA maps, as shown in Figure 3a2–d2, progressively dissipated into a homogeneous field by 400 h. Concurrently, iFFT images (Figure 3a3‐d3) revealed a reduction in fringe spacing from 0.203 to 0.166 nm. The corresponding intensity profiles (Figure 3a4–d4) evolved from relatively complex modulations at intermediate stages to a more regular single‐period oscillation after 400 h, consistent with progressive local relaxation and structural homogenization. Notably, embedded Cu‐rich regions progressively lost lattice coherence (Note S1).

**FIGURE 4:**
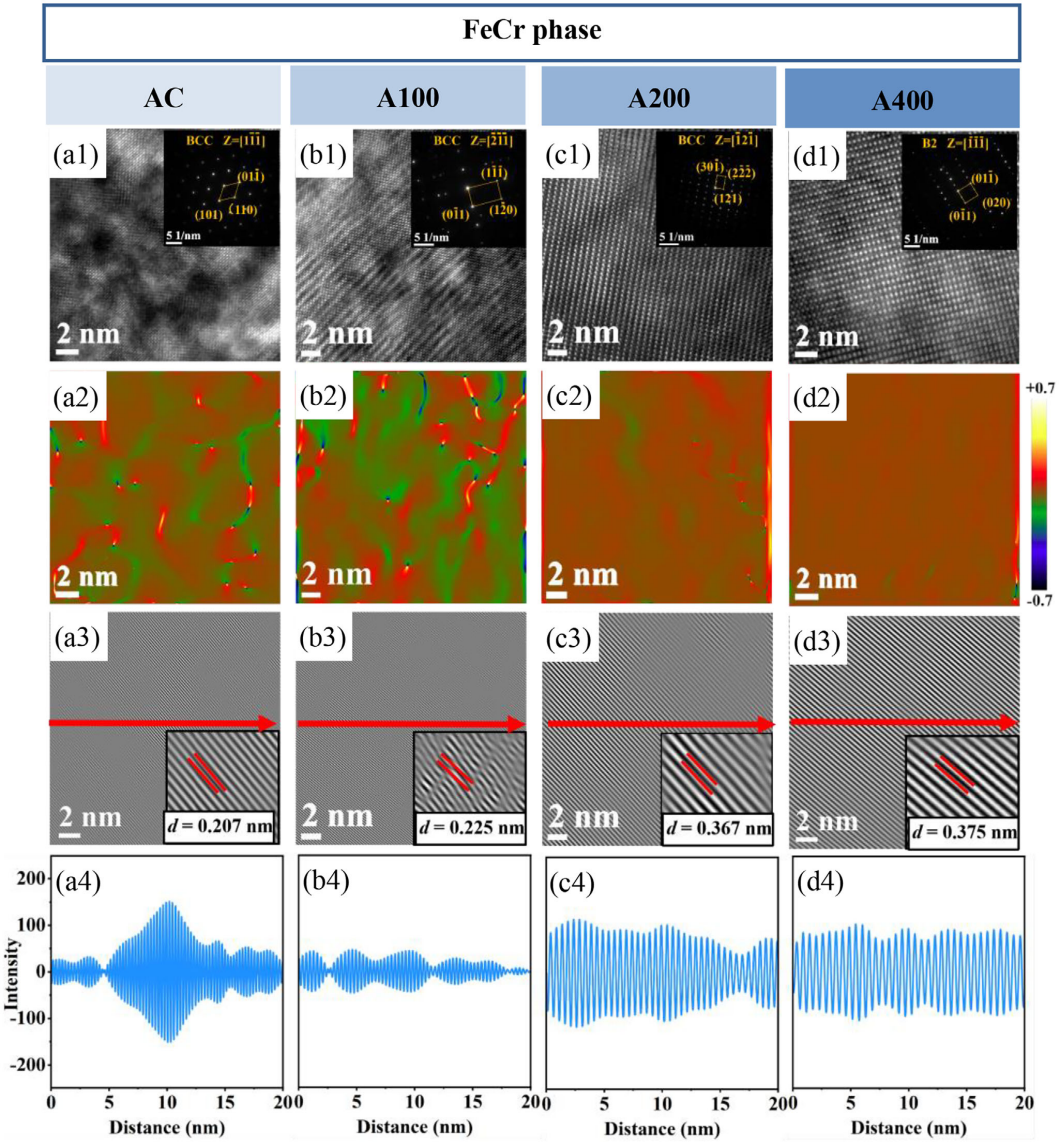
HR‐TEM images with corresponding SAED patterns, strain maps showing horizontal normal strain (ε_xx_), iFFT of the HR‐TEM pattern, and iFFT intensity profiles of the FeCr phase in EHEA samples after different annealing times: (a1–a4) AC sample, (b1–b4) A100 sample, (c1–c4) A200 sample, (d1–d4) A400 sample.

The FeCr phase progressively evolved from a fully disordered state to an ordered state (Figure 4). In the AC sample (Figure 4a1–a4), the phase was identified as being consistent with a disordered solid solution, as the SAED patterns displayed only fundamental BCC reflections. This correlated with qualitative observations of diffuse lattice contrast and pronounced local strain heterogeneity. Between A100 and A200 samples (Figure 4b1–c4), iFFT analysis revealed an increased fringe spacing from 0.225 to 0.367 nm alongside improved strain uniformity. However, the persistent absence of superlattice reflections indicated that no LRO had yet occurred. After 400 h of annealing (Figure 4d1–d4), clear evidence for long‐range B2‐type ordering was provided by the emergence of distinct (100) superlattice reflections in the SAED pattern. This transformation was visually and analytically supported by a highly homogenized strain field, uniform lattice fringes with a spacing of 0.375 nm, and an iFFT profile dominated by a strong single‐period oscillation.

## 3 Discussion

### 3.1 Phase Kinetic Evolution During Annealing

The structural evolution at 898 K proceeds via two distinct, asynchronous pathways. The Cu‐rich and B2‐NiAl phases undergo rapid compositional and strain redistribution during early‐stage annealing, whereas the FeCr phase exhibits a progressive development of lattice periodicity over an extended interval. This kinetic disparity suggests that as‐cast heterogeneities are accommodated through phase‐specific atomic redistribution mechanisms rather than a uniform structural response.

#### 3.1.1 Evolution of the Cu‑Rich and B2‑NiAl Phases

An endothermic peak was observed in the as‐cast state and disappeared by 200 h (Figure 1a,b), indicating early‐stage internal structural adjustments. While the B2‐NiAl phase retained its LRO structure during annealing, it underwent progressive local relaxation. This decoupled structural evolution is evidenced by GPA maps, as shown in Figure 3a1–d4, where localized high‐contrast strain visibly transitions into a homogeneous field. In embedded Cu‐rich regions, diffraction patterns (Figure S2) revealed a localized L1_2_‐to‐FCC transformation after 100 h of annealing that contributes to overall matrix homogenization. However, the transformation of these Cu‐rich features likely involves more complex coupled effects, including compositional evolution, interfacial effects, and size‐dependent thermodynamics, which are beyond the scope of the present phase‐resolved defect‐transport analysis [40]. Therefore, the mechanistic discussion in this work focuses on the B2‐NiAl and BCC‐FeCr phases as the primary carriers of the asynchronous ordering response.

#### 3.1.2 Evolution of the FeCr Phase

The FeCr phase followed a protracted ordering sequence as demonstrated by the sequential structural transitions in Figure 4a1–d4. In the AC sample, the initial state was consistent with a disordered solid solution based on the absence of superlattice reflections, while diffuse lattice contrast and localized strain variations visually reflected the inherent structural heterogeneity. Rather than an abrupt structural jump, the subsequent evolution proceeded through distinct intermediate states. In A100 sample, weak periodic modulations in the iFFT images emerged, signifying the development of transient short‐range correlations. This further progressed to a coherently correlated medium‐range periodic arrangement in A200 sample, where directionally aligned fringes and increased strain uniformity were observed. However, the continuous lack of superlattice spots during this period confirmed that long‐range chemical ordering was not yet detectable. LRO became clearly evident only after 400 h of annealing, as indicated by the emergence of (100) superlattice reflections and the accompanying structural homogenization. This staged evolution from a highly distorted solid solution to an LRO structure suggests that the FeCr phase accommodates thermal exposure through gradual, diffusion‐controlled atomic rearrangements. Unlike the B2‐NiAl and Cu‐rich phases that reached structural stability within 200 h, the FeCr phase continued to evolve over a much longer timescale. This kinetic disparity successfully explains the absence of signals in the DSC curves because the sluggishly established ordering is kinetically irreversible within the applied cooling rate. Consequently, the microstructural stability of the EHEA is strongly influenced by this phase‐dependent kinetic landscape.

### 3.2 Atomic Configurations and the Preferences in Elemental Distributions

**FIGURE 5:**
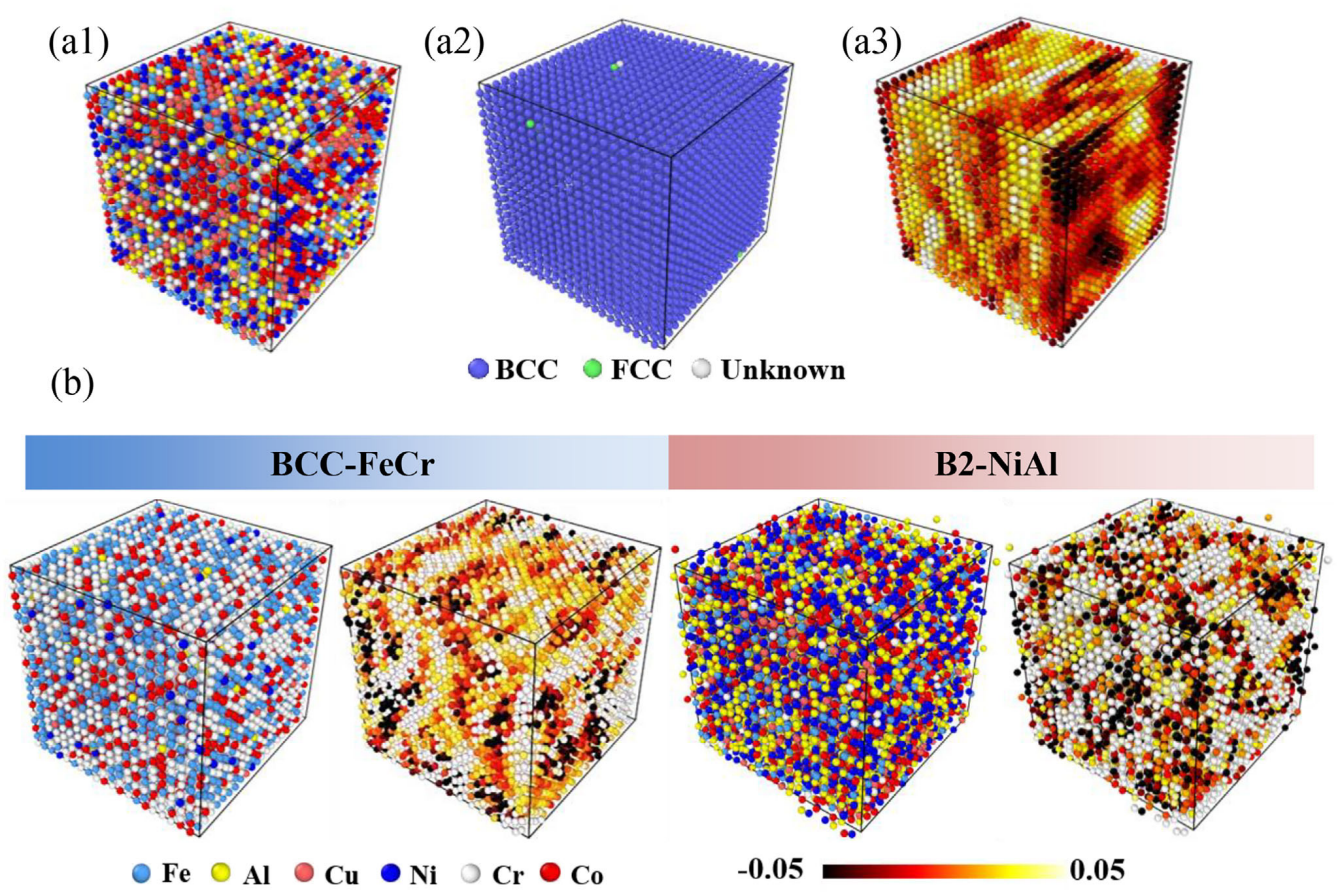
Atomic configurations and phase stabilities of the EHEA, (a1–a3) equiatomic AlCoCrCuFeNi composition, (b) BCC‐FeCr and B2‐NiAl phase compositions as listed in Table 1.

The atomistic configuration and energy minimization of equiatomic AlCoCrCuFeNi are illustrated in Figure 5a1. Energy minimization calculations in Figure 5a2 indicate that the BCC lattice provides a stable structural framework, while Figure 5a3 reveals pronounced local stress‐state heterogeneity originating from elemental segregation. This pronounced local stress heterogeneity, originating from elemental segregation, reflects a metastable state consistent with compositionally modulated phase separation [41]. Based on the compositions in Table 1, these stress‐strain fluctuations establish a heterogeneous structural background that dictates the subsequent diffusion‐controlled evolution in both BCC‐FeCr and B2‐NiAl phases.

Atomic mobility in EHEAs is inherently constrained by severe lattice distortions and electronegativity gradients, resulting in lower diffusion coefficients than conventional alloys [42]. Following spinodal decomposition, each phase develops distinct chemical compositions and lattice structures, which give rise to phase‐specific diffusion energy conditions. As illustrated in Figure 5b, the calculated diffusion energy barriers differ markedly between phases. The marked disparity in migration barriers suggests that atomic redistribution proceeds predominantly within individual phases rather than through extensive cross‐phase exchange. Specifically, while the ordered B2 structure offers relatively uniform diffusion pathways, the disordered BCC structure imposes tortuous paths characterized by a broad distribution of migration barriers [43, 44].

### 3.3 Atomic Transport in BCC‐FeCr Phase and B2‐NiAl Phase

**FIGURE 6:**
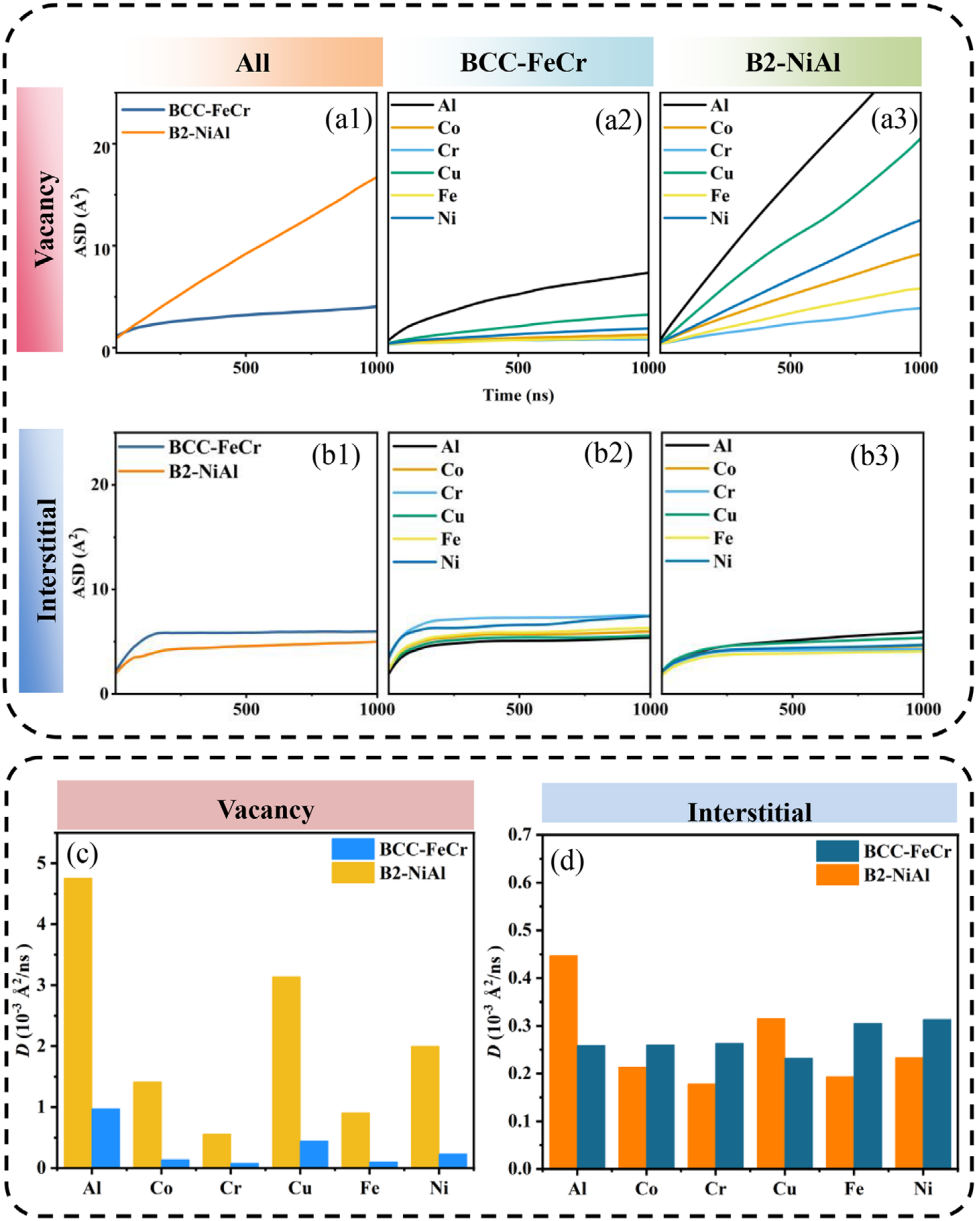
Atomic square displacement (ASD) and diffusion coefficients for vacancy and interstitial atoms in BCC‐FeCr and B2‐NiAl phases, (a1–a3) vacancy ASD in BCC‐FeCr and B2‐NiAl, (b1–b3) interstitial ASD in BCC‐FeCr and B2‐NiAl. (c) Vacancy diffusion coefficients in BCC‐FeCr and B2‐NiAl. (d) Interstitial diffusion coefficients in BCC‐FeCr and B2‐NiAl.

The atomic transport properties of the BCC‐FeCr and B2‐NiAl phases were investigated using MD simulations at 898 K to elucidate relative defect‐mediated transport tendencies. Fickian diffusion behavior was confirmed for vacancy transport through the linear regime of the atomic square displacement (ASD) curves, while interstitial diffusion exhibited an initial increase followed by a sub‐diffusive behavior in Figure 6a1–b3. For the BCC‐FeCr phase, both vacancy diffusion coefficient (3.75 × 10^−4^ Å^2^ ns^−1^) and interstitial diffusion coefficient (2.35 × 10^−4^ Å^2^ ns^−1^) remained of the same order of magnitude. This similarity indicates that both defect‐mediated processes contribute concurrently to atomic transport, which provides the necessary mobility for the disorder‐to‐order transition. Conversely, the vacancy diffusion coefficient in the B2‐NiAl phase (2.596 × 10^−3^ Å^2^ ns^−1^) exceeds the interstitial diffusion coefficient (2.25 × 10^−4^ Å^2^ ns^−1^) by an order of magnitude, suggesting that vacancy‐mediated transport is strongly favored in the ordered structure.

Element‐specific diffusivity was also evaluated, as shown in Figure 6c,d. Both phases exhibited a consistent hierarchy of vacancy diffusivity in the order of Al > Cu > Ni > Co > Fe > Cr, which identifies Al, Cu, and Ni as the primary contributors to vacancy‐mediated transport. While interstitial diffusion in the BCC‐FeCr phase was relatively uniform across all elements, Al and Cu displayed significantly higher interstitial diffusivities in the B2‐NiAl phase. These differences reflect phase‐dependent defect energetics rather than a uniform interstitial transport mechanism. These diffusion characteristics support distinct transport pathways that correlate with the microstructural evolution observed experimentally. The B2‐NiAl phase maintains its ordered structure through vacancy‐mediated diffusion, which involves highly mobile Al and Cu atoms that facilitate lattice adjustments while preserving LRO [45]. In contrast, the BCC‐FeCr phase is characterized by a heterogeneous energy landscape that constrains long‐range vacancy transport [46]. Under these conditions, cooperative vacancy‐interstitial transport is therefore proposed as a kinetic accommodation mechanism. This synergistic transport mitigates the limitations imposed by low vacancy populations and enables the thermodynamically driven ordering to proceed gradually in the BCC‐FeCr phase [47].

To elucidate the atomic‐scale diffusion mechanisms, vacancy formation and migration energies were calculated for both BCC‐FeCr and B2‐NiAl phases. Vacancy diffusion involves two key energetic parameters. Formation energy determines the equilibrium vacancy concentration, while migration energy governs the activation barrier for atomic jumps. The vacancy formation energy influences defect concentration via the Boltzmann relation, and the migration energy determines the thermal activation required for vacancy‐atom exchange. Based on this framework, vacancy formation energies for each element in the EHEA were calculated using the following equation.

(1) $$E_{f}=E_{V}-\frac{N-1}{N}E_{\mathrm{bulk}}$$

where *E_v_* represents the potential energy of a system containing one vacancy after energy minimization, and *E_bulk_* represents the energy of a perfect lattice. *N* is the number of atoms in the perfect lattice, thus *E_bulk_ /N* represents the potential energy per atom, equivalent to the chemical potential *µ*. Since the EHEA contains six elements, the average potential energy per atom is used as the reference chemical potential.

**FIGURE 7:**
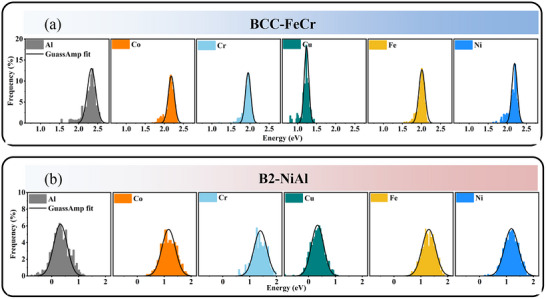
Vacancy formation energies for each element in BCC‐FeCr phase (a) and B2‐NiAl phase (b). The curve in each panel represents the best nonlinear fit according to the amplitude Gaussian peak function.

Figure 7a,b shows the vacancy formation energy distributions for each element in BCC‐FeCr and B2‐NiAl phases. The vacancy formation energies range from 1.23 to 2.6 eV in BCC‐FeCr phase and from 0.32 to 1.37 eV in B2‐NiAl phase. These distributions reflect the structural heterogeneity and spatial variations in the EHEA at atomic scale. For BCC‐FeCr phase, the full width at half maximum (FWHM) values indicate the heterogeneity order Al (2.33) > Ni (2.19) > Co (2.18) > Fe (2.01) > Cr (1.94) > Cu (1.23), indicating large spatial variations in local bonding environments. While low‐energy regions such as Fe‐rich and Cr‐rich clusters may facilitate vacancy formation, high‐energy regions may suppress it. These observations suggest the presence of heterogeneous vacancy distributions within the BCC phase, which likely limits vacancy‐dominated diffusion. Although local low‐energy regions can accumulate vacancies, the overall equilibrium vacancy concentration in BCC‐FeCr remains low, which may reduce the effectiveness of vacancy‐mediated diffusion.

The equilibrium vacancy concentration for both phases was obtained using the Boltzmann relation:

(2) $$n_{v}=\exp\left(-\frac{E_{f}}{k_{b}T}\right)$$

For B2‐NiAl phase, the heterogeneity order is Cr (1.37) > Fe (1.27) > Ni (1.16) > Co (1.14) > Al (0.32) = Cu (0.32). At 898 K (*k_B_T* = 0.078 eV), the lowest *E_f_* values are 1.23 eV (Cu) in BCC‐FeCr and 0.32 eV (Al, Cu) in B2‐NiAl. Substituting these values into the Boltzmann relation provides an upper‐bound estimate for vacancy concentration, indicating that the vacancy concentrations in the BCC‐FeCr and B2‐NiAl phases differ by more than five orders of magnitude. This difference suggests that vacancy‐dominated diffusion is more favorable in the B2‐NiAl phase, whereas the extremely low vacancy concentration in the BCC‐FeCr phase suggests that vacancy‐mediated transport alone may be insufficient to account for the observed atomic rearrangement, and that interstitial‐related processes may provide a supplementary kinetic contribution.

**FIGURE 8:**
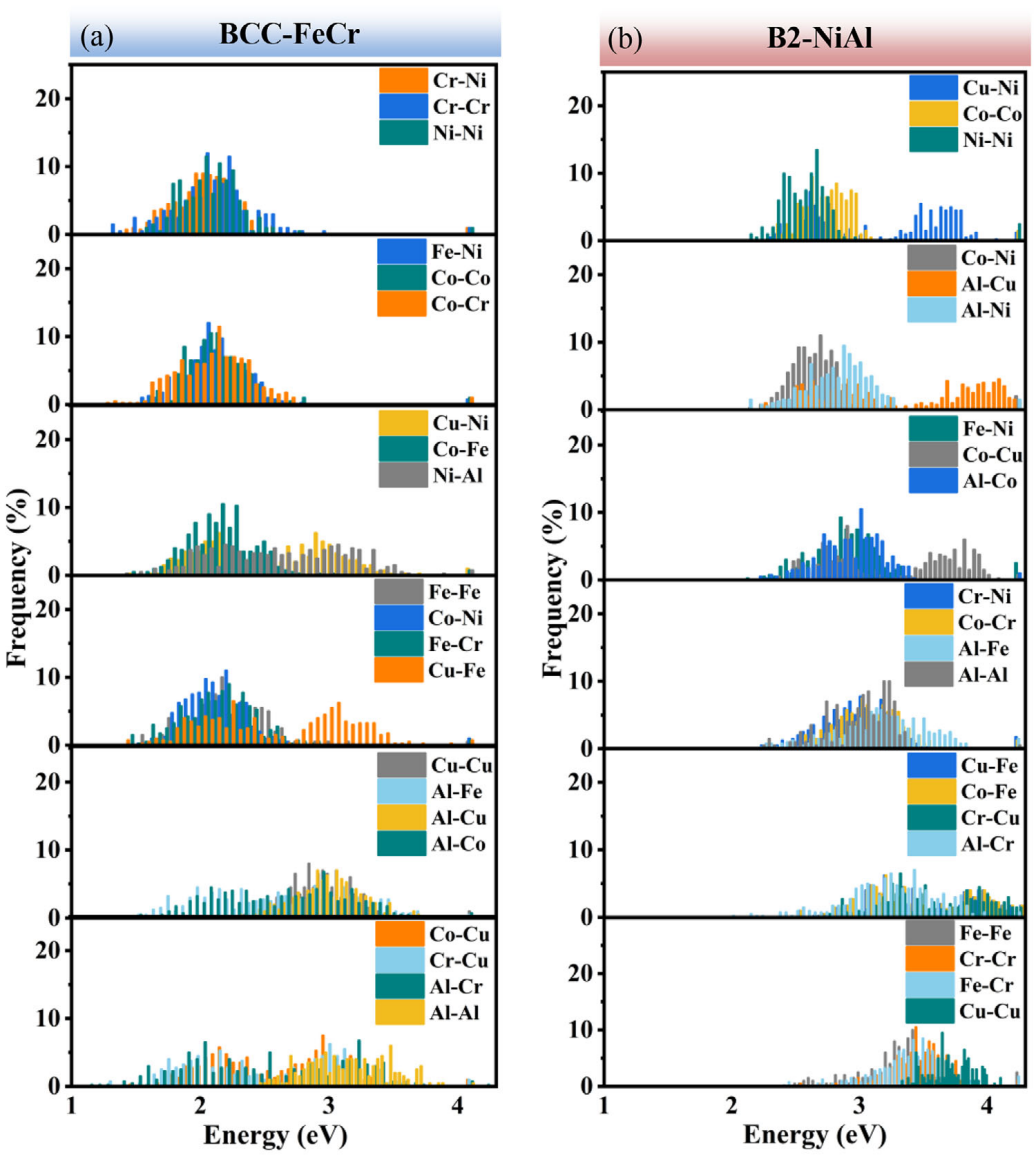
Formation energies of interstitial dumbbells in disordered BCC‐FeCr phase (a) and ordered B2‐NiAl phase (b).

Interstitial diffusion is another major point‐defect transport pathway. The interstitial formation energy of element atoms in EHEA was calculated by Equation (3).

(3) $$E_{f}=E_{i}-\frac{N+1}{N}E_{bulk}$$

where *E_i_* represents the potential energy of a model with one interstitial atom inserted (after energy minimization). Interstitial formation energies for different element dumbbells were calculated to understand the distinct interstitial diffusion behaviors in BCC‐FeCr and B2‐NiAl phases (Figure 8). In BCC‐FeCr phase (Figure 8a), Cr–Ni, Cr–Cr, and Ni–Ni dumbbells show formation energies of approximately 2.1 eV, which are significantly lower than other element combinations. This reduction in energy is attributed to the disordered atomic arrangement because irregular lattice sites and varied local chemical environments minimize the energy penalty for interstitial formation. These energetically favorable dumbbells are suggested to serve as intermediate states during atomic migration, which effectively lower migration barriers and increase the effective concentration of mobile interstitial defects within the BCC phase.

In contrast, the B2‐NiAl phase displays uniformly high interstitial formation energies exceeding 2.7 eV for all dumbbell combinations, with the Cu–Cu dumbbell reaching 3.8 eV (Figure 8b). The ordered B2 structure maintains regular sublattice arrangements that create high energy barriers against interstitial incorporation, which suppresses interstitial‐mediated diffusion [48]. This significant energetic disparity suggests that vacancy diffusion dominates in B2‐NiAl due to the inhibition of interstitial formation, whereas both vacancy and interstitial mechanisms contribute to transport in the BCC‐FeCr phase.

Heterogeneous interstitial energies and low vacancy concentrations, together with the results in Figure 6a2, indicate that migration in BCC‐FeCr is enabled by cooperative vacancy‐interstitial diffusion. Ordering is facilitated through defect migration to favorable sites. Conversely, defect generation is suppressed in B2‐NiAl by high interstitial formation energies, which ensures thermal stability. Vacancy diffusion remains the primary pathway for structural relaxation and LRO, while structural transitions are prevented by high barriers during high‐temperature treatment.

**FIGURE 9:**
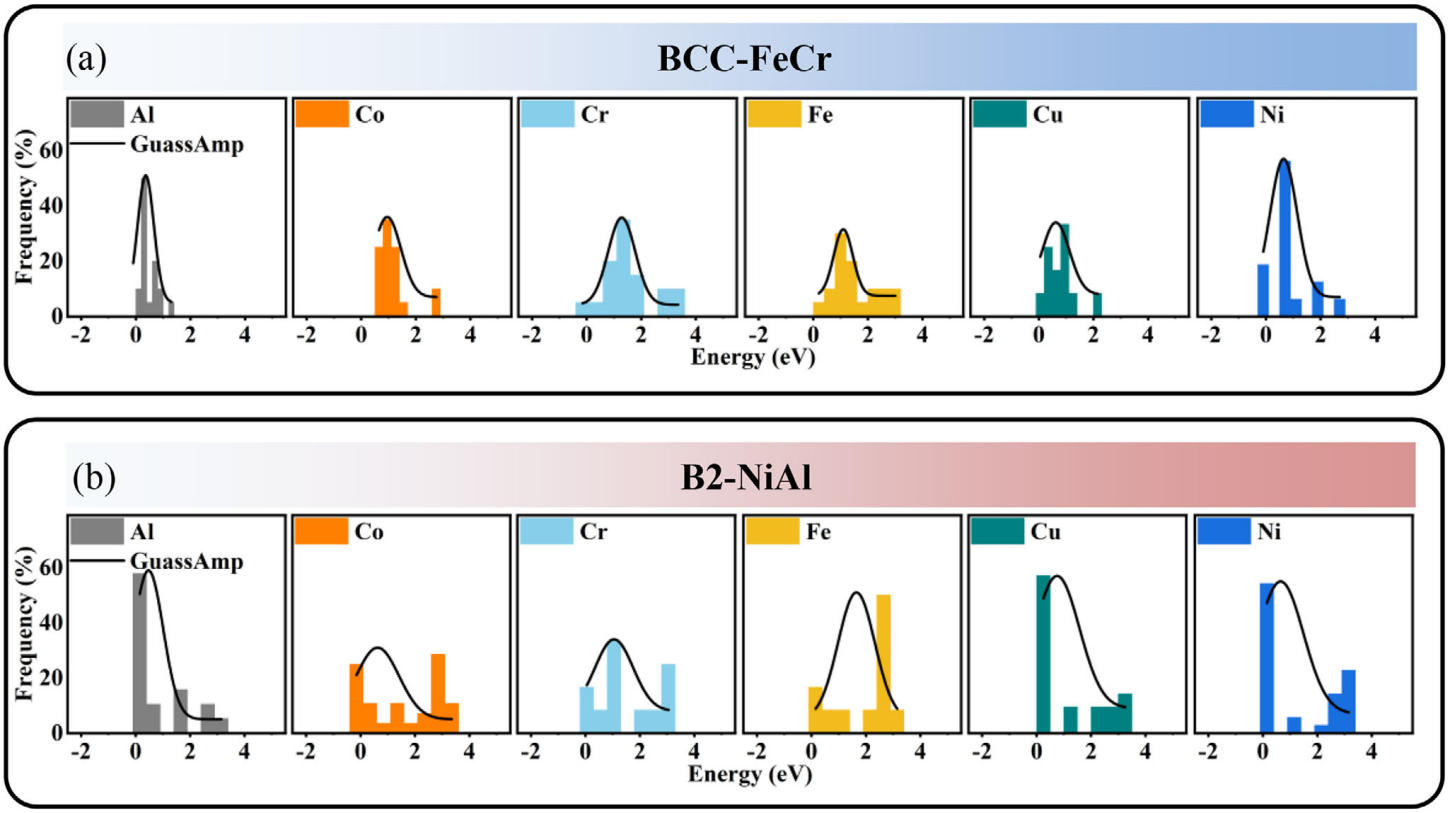
Spectra of vacancy migration energy for each element in BCC‐FeCr phase (a) and B2‐NiAl phase (b).

Vacancy migration energies were calculated for BCC‐FeCr and B2‐NiAl to clarify the microscopic origins of the observed diffusion behaviors. MD simulations confirmed that transport in both phases proceeds via nearest‐neighbor atomic exchanges. As illustrated in Figure 9a,b, migration energies in the BCC‐FeCr phase were found across a wide range from 0.47 to 1.65 eV. This dispersion reflects the heterogeneous local environments where variations in composition and lattice distortion generate a broad spectrum of exchange barriers. Consequently, vacancy‐mediated migration exhibits strong spatial variability, which is consistent with the limited long‐range diffusion efficiency observed in the ASD analysis. Conversely, migration energies in the B2‐NiAl phase were exhibited a narrower distribution between 0.36 and 1.28 eV. This narrower distribution reflects a uniform local environment imposed by the ordered lattice, which leads to consistent exchange barriers and higher diffusion coefficients as shown in Figure 6c. These energetics confirm that the ordered structure facilitates efficient vacancy transport, while the disordered BCC phase imposes significant constraints due to local chemical fluctuations.

**FIGURE 10:**
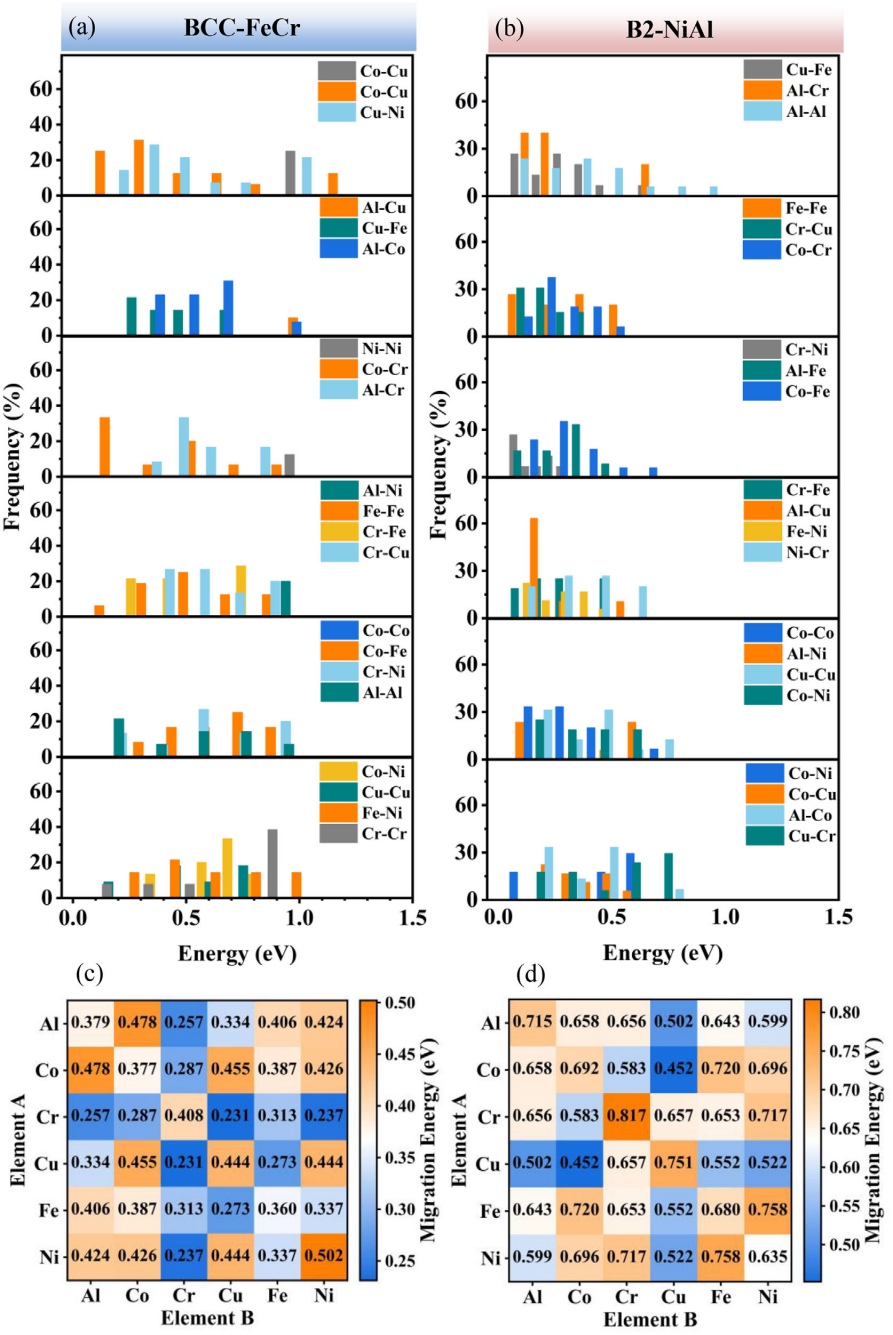
Distributions of migration energies for interstitial atoms in the BCC‐FeCr phase (a) and B2‐NiAl phase (b), and heatmaps of frequency‐weighted mean migration energies for atomic pairs within the BCC‐FeCr phase (c) and B2‐NiAl phase (d).

The microscopic origin of interstitial‐mediated transport was further examined through dumbbell migration energies (*E_m_*). In the BCC‐FeCr phase, the broad *E_m_* distribution of 0.10–1.15 eV (Figure 10a) indicates accessible local migration pathways in the disordered lattice. Frequency‐weighted mean migration energies for different atomic pairs (Figure 10c) range from 0.231 to 0.502 eV, with Cr–Cu, Cr–Ni, and Al–Cr being relatively low, indicating strongly pair‐dependent but overall accessible interstitial migration. The B2‐NiAl phase exhibits a higher *E_m_* range of 0.45–0.85 eV, as shown in Figure 10b, reflecting suppression of low‐energy interstitial pathways by the ordered lattice. Its frequency‐weighted mean migration energies are also higher overall, ranging from 0.452 to 0.817 eV (Figure 10d). Overall, interstitial transport is more accessible in BCC‐FeCr, whereas interstitial‐mediated migration in B2‐NiAl is more constrained.

The thermodynamic driving forces behind the microstructural evolution were analyzed through SRO parameters. The simulations showed that strong attractive interactions, such as Ni–Al and Cu–Cu, contribute to the stabilization of the B2‐NiAl phase, while repulsive interactions, such as Cu–Fe, discourage Cu incorporation into the matrix, supporting the dissolution of Cu–rich regions. These findings indicate that atomic redistribution during annealing is governed by a balance of attractive and repulsive forces, which drive the phase evolution observed experimentally. For more detailed SRO data and its implications on phase evolution, refer to Note S2.

### 3.4 Design Insights From Asynchronous Phase Evolution

The asynchronous evolution identified here provides a useful mechanistic basis for the design of eutectic alloys and related multiphase HEA/EHEA systems. In the present alloy, the B2‐NiAl phase responds relatively rapidly during annealing, whereas the BCC‐FeCr phase evolves more gradually toward ordering. This phase‐specific kinetic contrast suggests that asynchronous evolution can be exploited in heat‐treatment design: early annealing may be used to stabilize the fast‐responding phase and reduce metastable heterogeneity, whereas additional treatment may be introduced only when further evolution of the slower phase is desired. More broadly, composition design may target not only phase constitution, but also the kinetic contrast between coexisting phases through elemental partitioning and defect‐transport characteristics. In this way, asynchronous evolution offers guidance for designing eutectic alloys with both high thermal stability and tunable microstructural evolution.

## 4 Conclusion

In summary, the asynchronous ordering behavior of the AlCoCrCuFeNi eutectic high‐entropy alloy arises from fundamentally different defect‐transport mechanisms operating in its coexisting phases. The ordered B2‐NiAl phase accommodates annealing predominantly through efficient vacancy‐mediated transport, enabled by low vacancy formation and migration energies and a relatively uniform local environment. This transport mode supports local structural refinement and progressive strain relaxation while preserving the long‐range ordered framework. In contrast, the BCC‐FeCr phase undergoes a much slower disorder‐to‐order transition because vacancy‐mediated transport is strongly constrained by spatially heterogeneous defect energetics. Under these conditions, accessible low‐energy interstitial configurations provide an additional kinetic pathway, giving rise to cooperative vacancy‐interstitial transport that facilitates gradual atomic rearrangement and eventual long‐range ordering. These findings demonstrate that the sequence, timescale, and pathway of phase evolution in eutectic high‐entropy alloys are governed by phase‐specific defect energetics rather than by an averaged diffusion response. More broadly, this work establishes a phase‐resolved mechanistic framework for understanding thermal stability in complex concentrated alloys and offers guidance for designing multiphase alloys with controllable ordering behavior and improved high‐temperature stability.

## 5 Methods

### 5.1 Sample Preparation and Characterization

The button‐shaped EHEA ingot composed of AlCoCrCuFeNi (with equal atomic percentages of 16.6%) and weighing 100 g was fabricated by arc melting using high‐purity metals (purity exceeding 99.98%) in a protective argon atmosphere. Prior to melting, all elemental constituents were cleaned. Subsequently, the ingot underwent homogenization at 1373 K for 10 h within evacuated quartz ampules, followed by slow cooling to room temperature. The homogenized EHEA ingots were subjected to isothermal annealing at 898 K in a vacuum tube furnace. The annealing temperature was selected based on DSC measurements (Figure 1a), which revealed an endothermic event associated with structural adjustment. This temperature was therefore adopted to capture the corresponding structural evolution. The EHEA ingots were then processed for subsequent tests using electric spark wire cutting in accordance with experimental requirements.

Microstructures were analyzed using a scanning electron microscope (Zeiss Supra 55 VP FEG, Carl Zeiss AG, Germany) with an OXFORD X‐Max energy‐dispersive X‐ray spectrometer. Rietveld refinements of the XRD results were conducted using the GSAS‐II software. Foils for TEM were prepared by grinding 3 mm discs to a thickness of 50 ± 5 µm and then thinned to perforation through twin‐jet electropolishing using a mixed solution of 10% perchloric acid and 90% ethanol. TEM investigations were conducted on a JEOL JEM 210 F FEG‐TEM with a JEOL JED 230 T energy‐dispersive spectrometer (EDS) at 200 kV to capture SAED, dark‐field, and bright‐field images, and HR‐TEM images. Phase compositions were determined by TEM‐EDS point analysis using at least five measurements for each phase in each sample. Phase fractions were estimated from representative bright‐field TEM images by area‐fraction analysis. Minor phases were omitted from Table 1 when not detected in the analyzed TEM fields and SAED patterns.

### 5.2 Molecular Dynamics and Monte Carlo Calculations

MD simulations of thermally activated vacancy diffusion and interstitial atom migration in EHEAs were used to probe equilibrium elemental partitioning and ordering tendencies under fixed temperature conditions using LAMMPS [49]. Interatomic interactions were described by a custom‐fitted embedded atom method (EAM) potential developed in this work for the Al‐Co‐Cr‐Cu–Fe–Ni system. The potential follows the standard EAM formalism [50, 51, 52]. Details of the potential validation are provided in Note S3. Simulations were performed on 20 × 20 × 20 BCC supercells containing 16 000 lattice sites with a time step of 1 fs. Each system was first equilibrated in the NPT ensemble using a Nosé–Hoover thermostat and barostat to achieve thermal and mechanical equilibrium, followed by diffusion analysis in the NVT ensemble at the target temperature [53, 54]. Simulations, tailored to defect type (vacancy or interstitial), composition, crystal structure, and temperature, ran for at least 1 ns, which is sufficient to reach the diffusive regime for analysis. The tracer diffusion coefficients were computed at 898 K. This temperature corresponds to the experimental annealing condition, and the diffusion behavior was evaluated directly from the atomic mean‐square displacement at this temperature. Prior to computing the ASD of all atoms [55], each model was equilibrated at the target temperature for sufficient thermalization. Subsequently, the tracer diffusion coefficient (D*) was determined from the ASD data using the Einstein relation [56].

(4) $$D^{*}=\frac{\langle r_{S}^{2}\left(t\right)\rangle }{2nt}$$

where in $\left\langle r_{S}^{2}\left(t\right)\right\rangle$, *S* represents the mean squared displacement, *t* is the elapsed time, and *n* denotes the system dimensionality (*n* = 3 for three‐dimensional diffusion). The tracer diffusion coefficients were calculated using Equation (4). The Arrhenius relation in Equation (5) was introduced to describe the general temperature dependence of diffusion coefficients, while diffusion behavior in the present work was primarily analyzed at 898 K to enable direct comparison with experimental annealing conditions:

(5) $$D^{*}\;\left(T\right)=D_{0}\;exp\left(-E_{a}/k_{B}T\right)$$

To elucidate the mechanism underlying elemental partitioning in the EHEAs, defect formation was systematically analyzed by *E_f_* = *E_d_*  − *E_p_* ±_*d*_, where E_d_ and E_p_ are the total energies of the defect‐containing and perfect supercells, respectively, and µ_d_ is the chemical potential of the removed or added atom. In this study, µ_d_ was referenced to the average potential energy per atom of the corresponding phase, enabling consistent comparison of defect energetics between phases.

The migration energy of defects was assessed using the climbing image nudged elastic band (CI‐NEB) method within LAMMPS [57]. The CI‑NEB calculations were performed using the conjugate‐gradient minimization method with 10 intermediate images. The force convergence threshold for the NEB optimization was set to 0.01 eV Å^−1^. Pair‐resolved migration‐energy heatmaps were generated by calculating the frequency‐weighted mean migration energy for each atomic pair from its migration‐energy distribution. The weighted mean values were arranged into a symmetric matrix according to the six constituent elements and plotted as heatmaps to highlight the relative migration tendency of different atomic pairs within each phase.

To further clarify the elemental distribution trends in EHEAs, MD simulations were combined with the classical MC simulations using the conjugate gradient algorithm to relax the system toward its lowest potential energy state. Elemental swaps were accepted according to the thermodynamic probability at a given temperature *T*, specifically when *ΔE* < 0, based on the acceptance criterion exp (‐*ΔE* /*k_B_T*), where *k_B_* is the Boltzmann constant. Typically, approximately 1 000 000 MC steps were performed, and equilibrium was considered achieved when the system energy reached a minimum under the specified conditions. The simulations focus on phase‐to‐phase contrasts in defect energetics rather than absolute defect energies.

During the modeling process, the chemical ordering of the system was monitored in real time by evaluating both SRO and LRO parameters. The Warren–Cowley SRO parameter is defined as [58]:

(6) $$\alpha_{k}^{ij}=1-\frac{p_{k}^{ij}}{c_{i}}$$

where $p_{k}^{ij}$ is the conditional probability of finding an atom of type *i* in the *k*_th_ neighbor shell of atom *j*, and *c_i_* is the concentration of species *i*. $\alpha_{ij}^{k}=0$ indicates a random atomic distribution, while deviations from zero signify chemical ordering or segregation.

## Author Contributions

Huiwen Yao conducted the investigation, curated the data, and prepared the original draft of the manuscript. Qingshuang Ma was responsible for the conceptualization of the study and provided supervision, as well as reviewing and editing the manuscript. Jie Xiong and Jing Bai contributed to the investigation and data curation. Huijun Li contributed to the development of the methodology. Qiuzhi Gao supervised the study, acquired funding, and participated in the review and editing of the manuscript. All authors contributed to the interpretation of the results and approved the final version of the manuscript.

## Conflicts of Interest

The authors declare no conflicts of interest.

## Supporting information




**Supporting File**: advs75539‐sup‐0001‐SuppMat.docx.

## Data Availability

The data that support the findings of this study are available from the corresponding author upon reasonable request.
