# Supplementary material for: Phase‐Resolved Defect Transport Mechanisms Governing Asynchronous Ordering in a Eutectic High‐Entropy Alloy
Source: Adv Sci (Weinh). 2026 May 7;13(42):e75539. doi: 10.1002/advs.75539 (PMC13335875; doi:10.1002/advs.75539)
Supplement: Supplementary file 1 — Supporting File: advs75539‐sup‐0001‐SuppMat.docx. [file ADVS-13-e75539-s001.docx]

**Supporting Information for**

**Phase-resolved defect transport mechanisms governing asynchronous ordering in a eutectic high-entropy alloy**


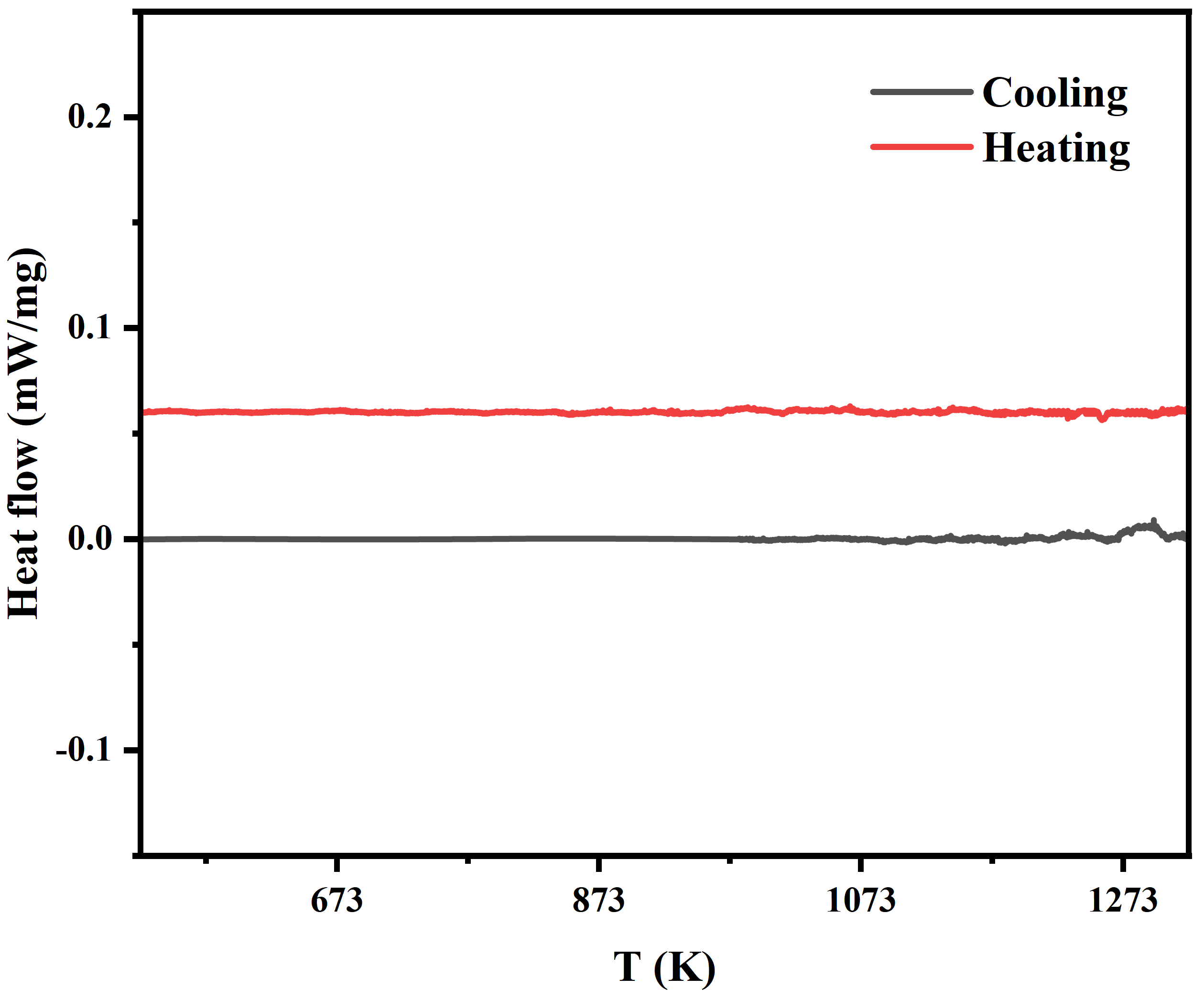

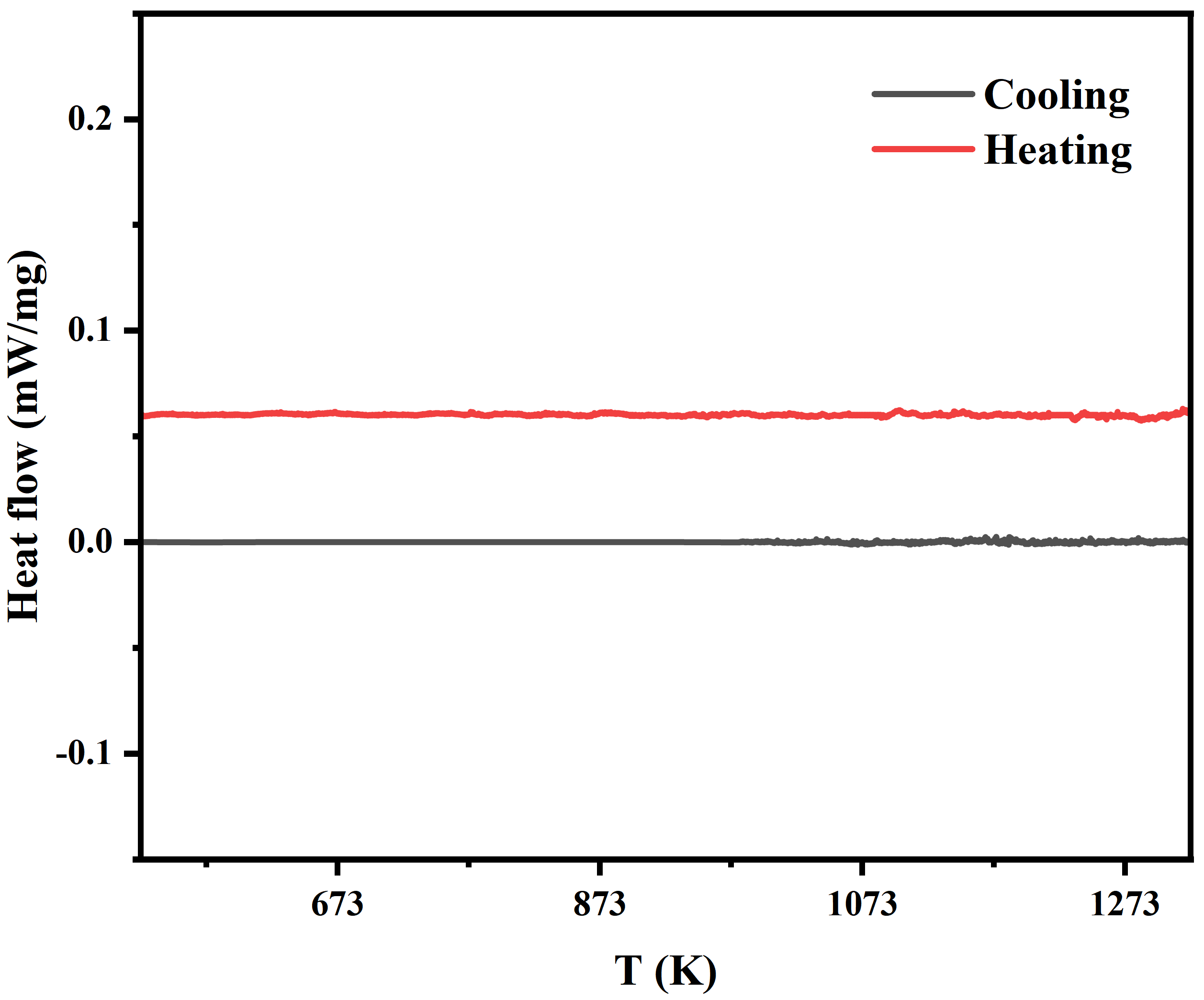

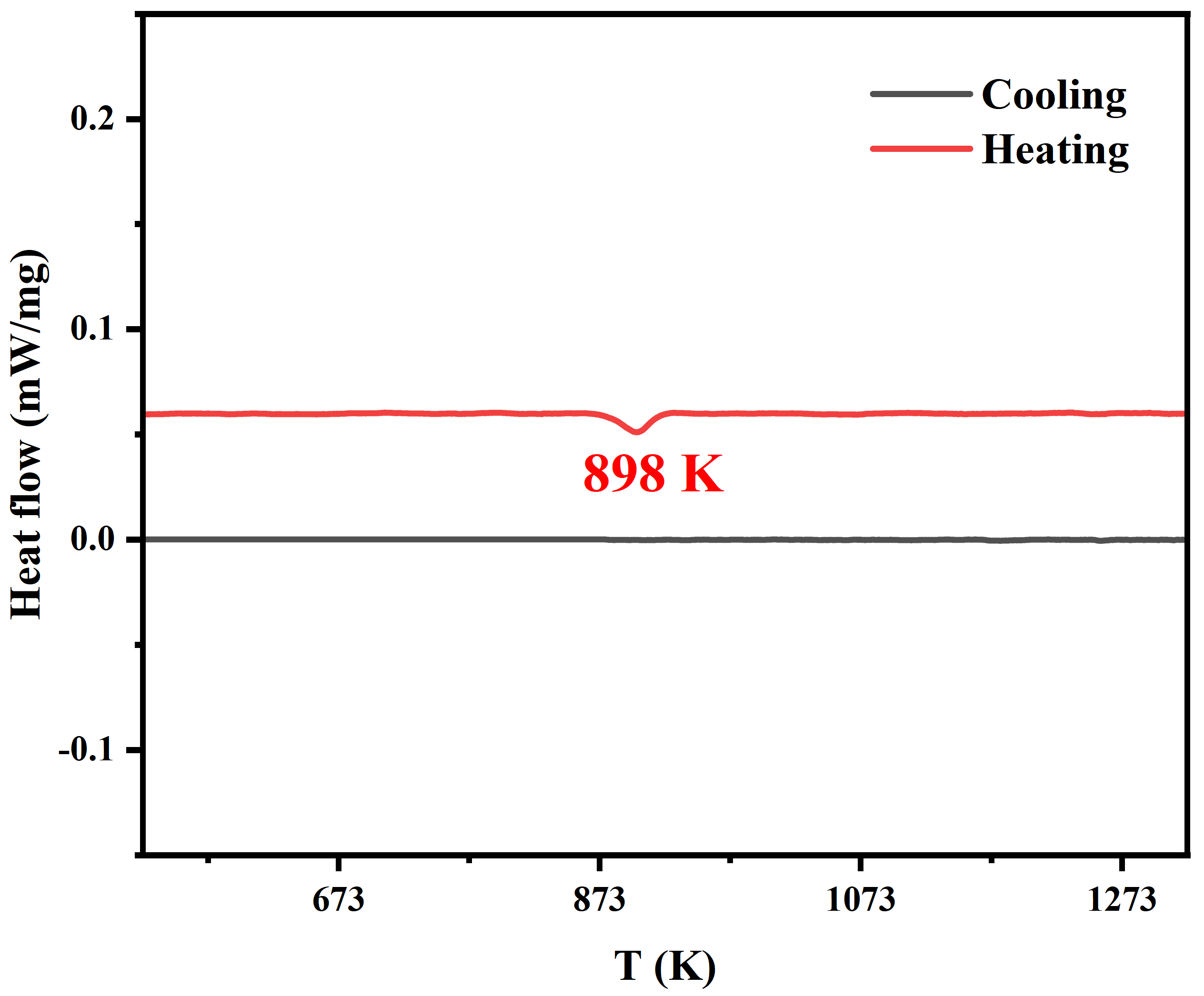


**A100**

**A200**

**A400**

(a)


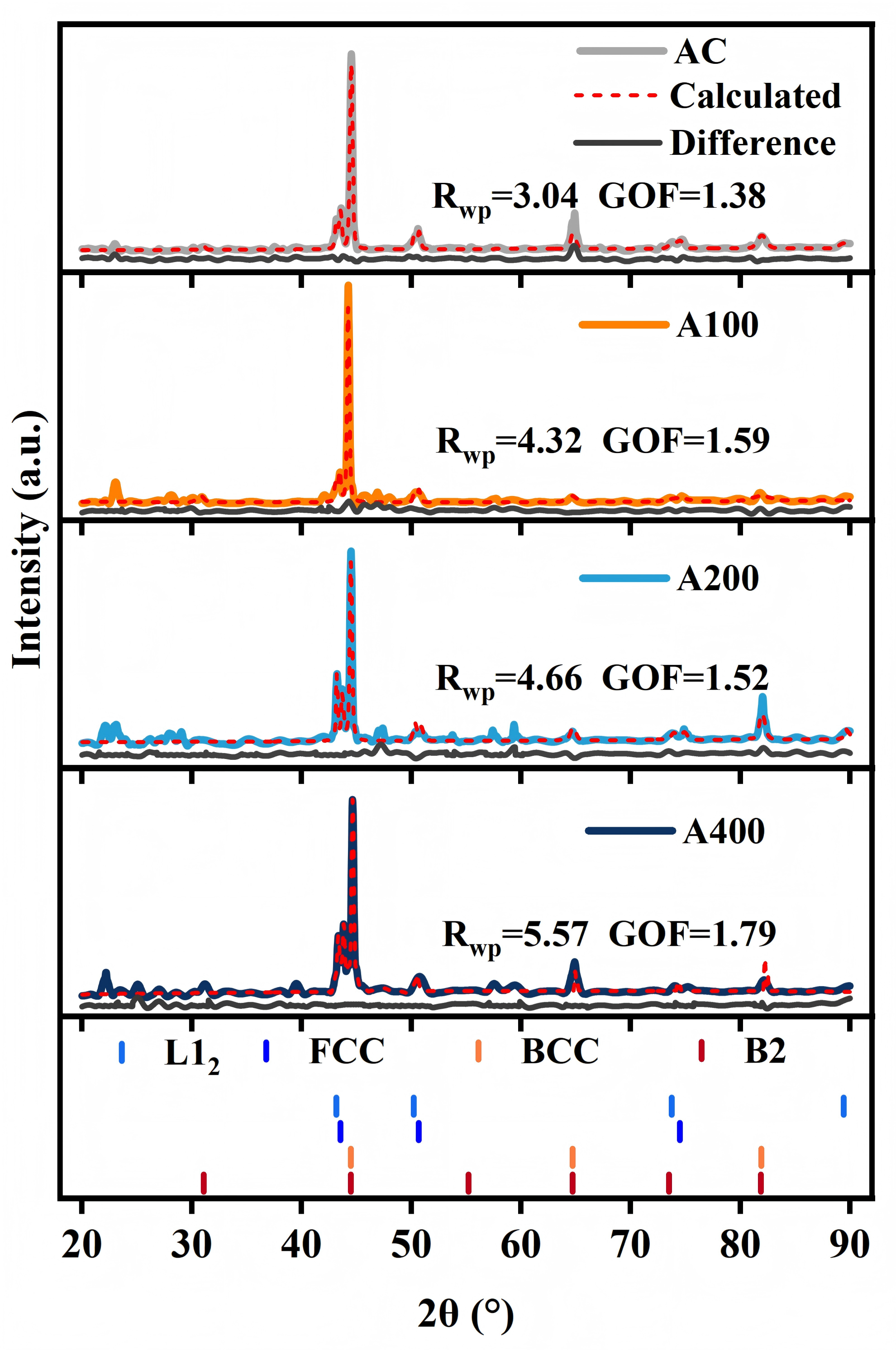


(b)

Fig. S1 DSC heating and cooling curves (a) and Rietveld refinement profiles of the XRD patterns (b) for the AlCoCrCuFeNi EHEA samples after different annealing times.

**Note 1: Cu-rich phase structural evolution**

Fig. S2 presents the atomic-scale characteristics of the Cu-rich phase at different annealing durations, highlighting the evolution of lattice contrast and periodic features. In the AC sample (Fig. S2(a1)), the Cu-rich region exhibits relatively uniform lattice contrast without pronounced directional alignment. In Fig. S2(a2), the corresponding inverse Fourier transform (iFFT) reveals fine, closely spaced periodic fringes, and the intensity profile Fig. S2(a3) is dominated by a single principal periodic component with low-amplitude oscillations, indicating a high degree of structural regularity. After 100 h annealing (Fig. S2(b1)) the Cu-rich phase shows increased contrast heterogeneity, accompanied by coarser and less sharply defined iFFT fringes (Fig. S2(b2)). The intensity profile (Fig. S2(b3)) retains a dominant periodic feature, while a modest increase in oscillation amplitude and a broadened envelope are observed, suggesting the emergence of additional weak periodic contributions. With further annealing to 200 h (Fig. S2(c1)), the lattice contrast exhibits more pronounced directional characteristics, and the iFFT fringes appear more continuous but less uniformly periodic in Fig. S2(c2). The corresponding intensity profile (Fig. S2(c3)) maintains an oscillatory nature; however, the coexistence of multiple periodic components leads to reduced regularity of the dominant periodic signal. Overall, the Cu-rich phase displays a progressive weakening of periodic coherence and increasing structural heterogeneity with annealing time, as reflected by the evolution of lattice contrast, iFFT fringe continuity, and intensity modulation.


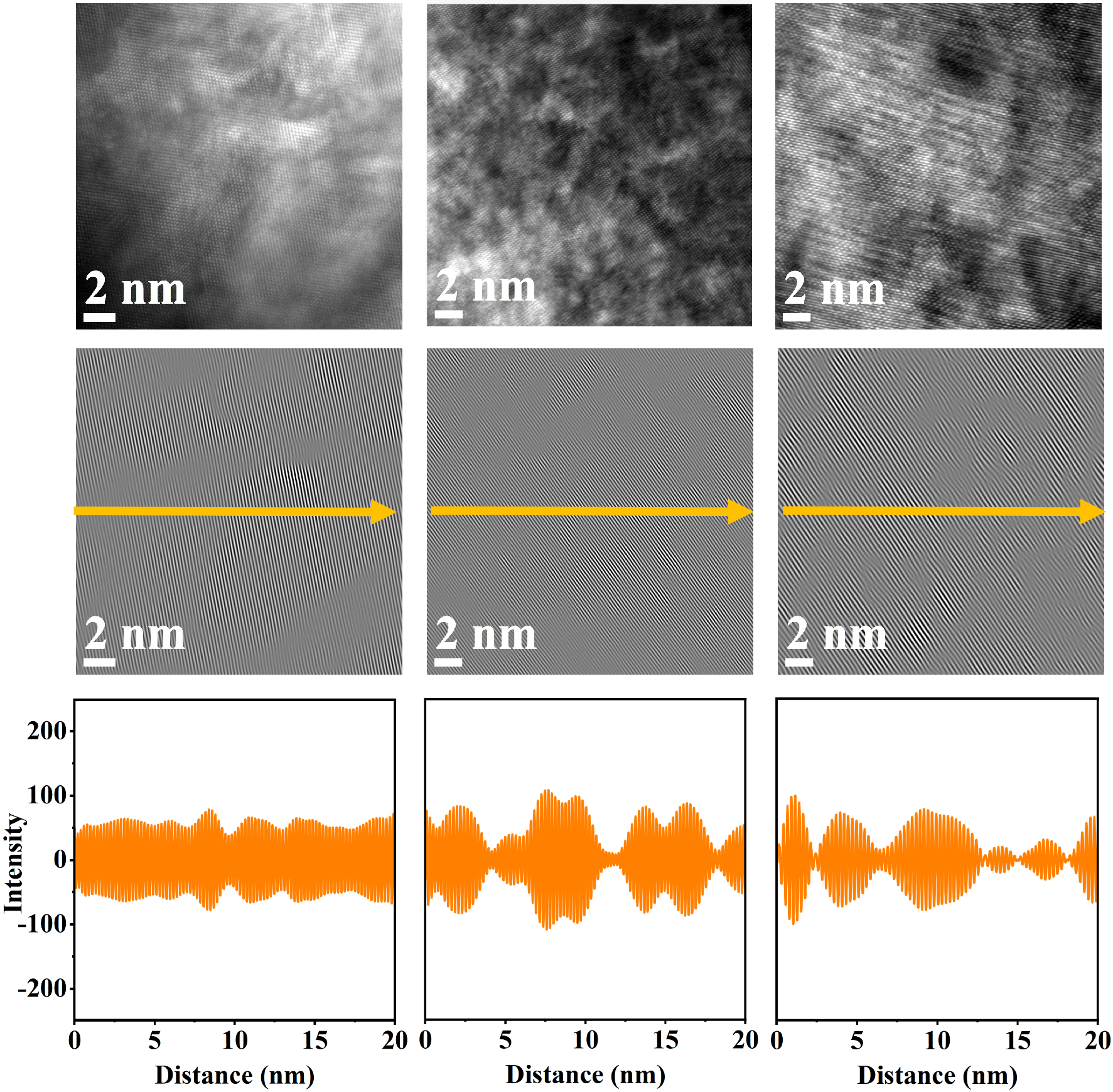


**AC**

**A100**

**A200**

(a1)

(b1)

(c1)

(a2)

(a3)

(b2)

(b3)

(c2)

(c3)

**Cu-rich Phase**

Fig. S2 HR-TEM with the iFFT of the HR-TEM, pattern, the intensity of iFFT of Cu-rich phase in EHEA samples: (a1)-(a3) AC sample, (b1)-(b3) A100 sample, (c1)-(c3) A200 sample.

**Note 2: Short-range order and thermodynamic interaction hierarchy**

The thermodynamic driving forces for microstructural evolution are quantified by the SRO parameters of the constituent atomic pairs. MC simulations performed at 898 K reveal the evolution of atomic configurations, where the total potential energy decreases with increasing MC steps, signifying the gradual formation of energetically more stable arrangements. This energetic relaxation reflects the development of a more stable compositional distribution, as further elucidated by the SRO parameters are shown in Fig. S3.

Analysis of SRO parameters reveals a distinct hierarchy of atomic interaction propensities. In Fig. S3(a), atomic pairs exhibiting strong attractive interactions (negative SRO) include Cu-Cu (-1.72), Ni-Al (-0.99), Ni-Ni (-0.40), Cr-Fe (-0.37), Co-Fe (-0.36), Al-Cr (-0.30), and Co-Al (-0.29). These strong thermodynamic affinities are consistent with the experimentally observed tendency toward the formation and stabilization of the B2-NiAl phase, driven by Ni-Al and Ni-Ni attractions, as well as the BCC-FeCr phase (influenced by Cr-Fe, Co-Fe, Al-Cr, Al-Fe attractions). Conversely, strong repulsive interactions (positive SRO) are evidenced by Cu-Fe (0.83), Al-Cu (0.45), Co-Cu (0.33), Cu-Ni (0.30), and Cr-Cu (0.52). The strong chemical incompatibility between Cu and multiple matrix elements provides a thermodynamic preference against Cu incorporation, which thermodynamically favors the dissolution of metastable Cu-rich FCC regions and their redistribution into more stable surrounding phases, particularly the B2-NiAl matrix, in conjunction with diffusion processes during annealing. Other pairs in Fig. S3(b), such as Al-Al (0.17), Fe-Fe (0.14), Co-Ni (0.04), and Cr-Ni (0.03), exhibit comparatively weaker, near-random SRO. The disparity in SRO magnitudes and the nature of these atomic interactions define the distinct thermodynamic conditions of each phase. Strong attractive SROs (like Ni-Al, Cu-Cu) establish a potent thermodynamic drive for rapid structural finalization in regions like B2-NiAl. In contrast, the BCC-FeCr phase, while also exhibiting some attractive SROs, is simultaneously influenced by strong repulsive interactions (such as Fe-Cr, Fe-Cu), suggesting a more complex and energetically frustrated ordering pathway.

The observed asynchronous evolution may be interpreted within a hierarchical thermodynamic landscape defined by the SRO parameters. Variations in the magnitude and sign of the SRO values reflect differences in chemical interaction preferences among atomic pairs, suggesting unequal thermodynamic tendencies for local ordering. These tendencies provide a thermodynamic background that, in combination with diffusion-controlled processes discussed earlier, may contribute to the staggered structural evolution observed during isothermal annealing. The present SRO analysis therefore serves as a qualitative thermodynamic reference rather than a quantitative predictor of kinetic behavior.

(a)

(b)


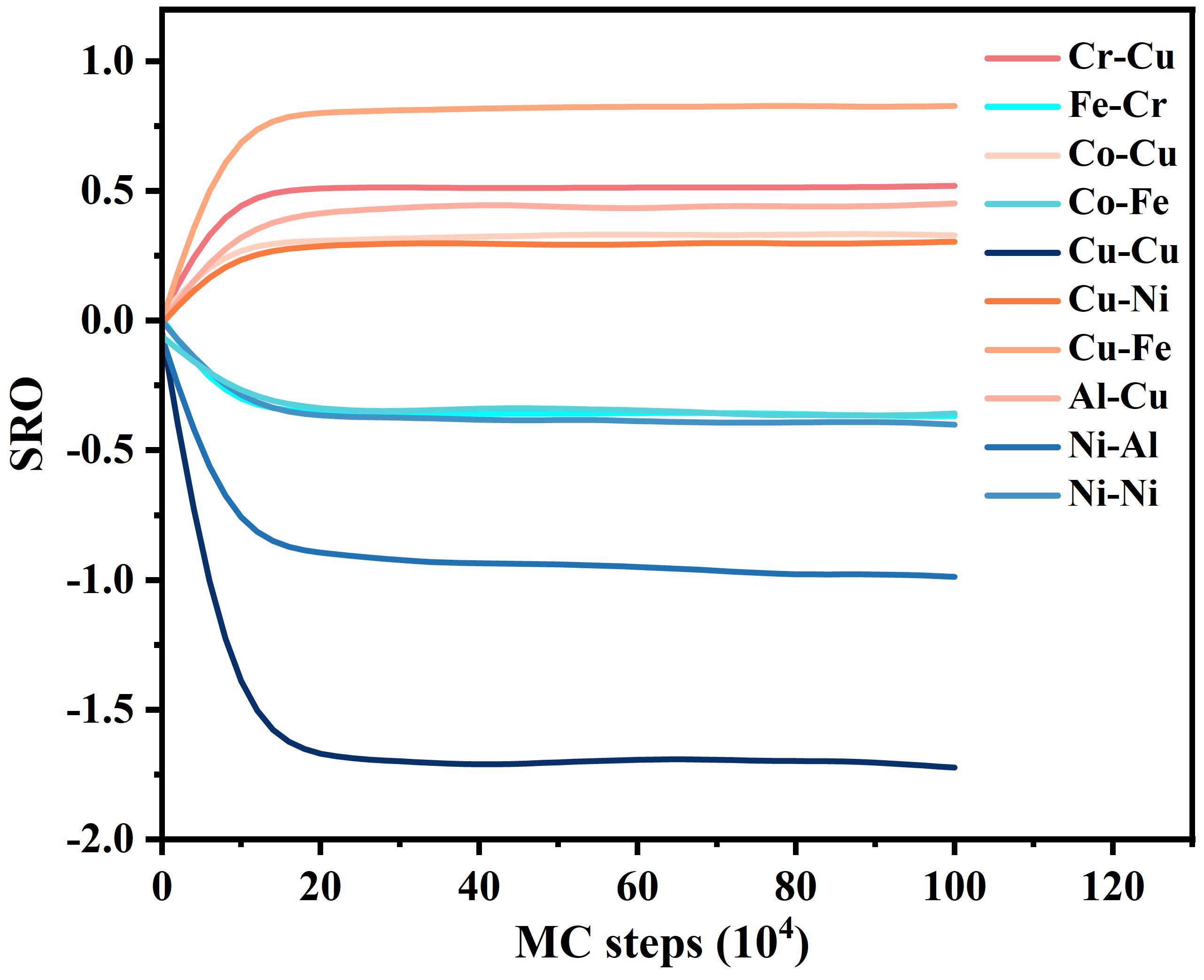

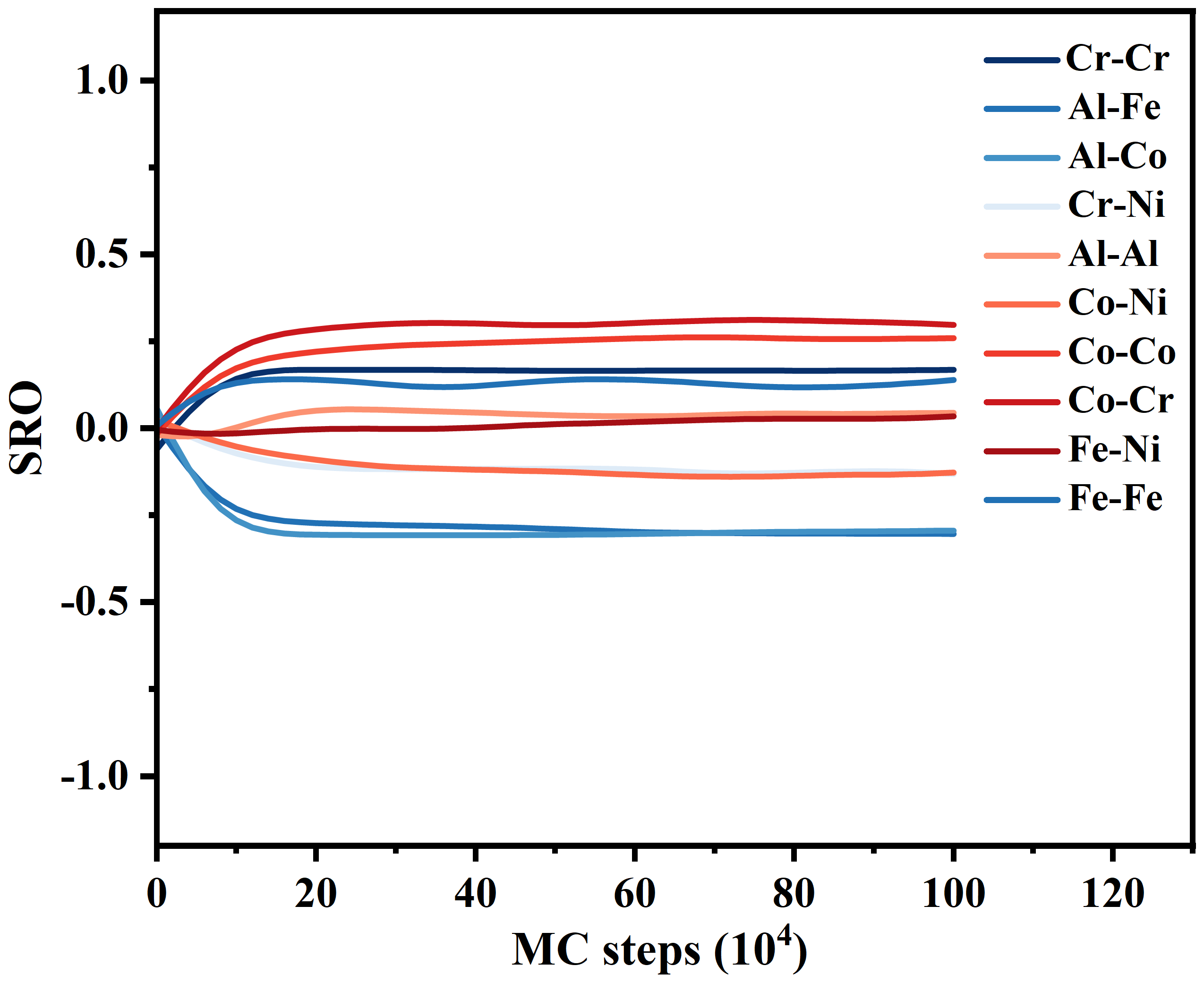


Fig. S3 (a) Short-range order parameters for key atomic pairs during MC equilibration. (b) Other SRO parameters for all atomic pairs.

**Note 3: Validating the reliability of the atomic potential**

To evaluate the reliability of the Co-Ni-Cr-Fe-Al-Cu EAM interatomic potential used in this study, we conducted three independent validations:

(1) Lattice constants,

(2) Cohesive energies,

(3) The melting point.

The lattice constants of the AlFeNiCoCrCu EHEA phases were experimentally measured by TEM. The BCC and B2 phases exhibit lattice constants of 2.86 Å, while the L1_2_ phase shows a lattice constant of 3.63 Å. The compositions of different phases determined by TEM-EDS analysis are BCC-FeCr (Cr_0.50_Fe_0.29_Co_0.16_Ni_0.24_Al_0.13_Cu_0.07_), B2-NiAl (Ni_0.30_Al_0.29_Co_0.19_Fe_0.12_Cu_0.06-_Cr_0.03_), and L1_2_ (Cu_0.91_Al_0.04_Ni_0.03_Co_0.005_Fe_0.005_). Molecular dynamics simulations were performed using these measured compositions, yielding lattice constants of 2.84 Å, 2.86 Å and 3.65 Å for BCC-FeCr, B2-NiAl and L1_2_ phases, respectively. The cohesive‑energy–lattice‑constant relationships shown in Fig. S4 confirm that the predicted equilibrium lattice constants correspond to the minimum cohesive‑energy states. The simulated values agree well with the experimentally measured lattice constants, indicating that the EAM potential captures the correct atomic‑scale structural characteristics.

To further assess the predictive capability of the EAM potential, we compared the cohesive energies of all Al‑containing B2 binary compounds formed from {Al, Co, Cr, Cu, Fe, Ni}, calculated using both density‑functional theory (DFT) and the EAM potential. The results, summarized in Table S1, show consistent energetic trends between DFT and EAM. For example, both methods identify NiAl as having strong cohesive energy (-2.422 eV/atom EAM and -2.575 eV/atom DFT), FeAl pairs show similar behavior (-2.482 eV/atom EAM and -2.582 eV/atom DFT), while CuAl exhibits the weakest cohesion among the B2 compounds. Deviations between the two datasets range from 1.66% to 6.31%, demonstrating good quantitative agreement.

We also simulated the melting behavior of AlFeNiCoCrCu EHEA by heating the system from 300 K to 2000 K at 10 K/ps. The potential‑energy curve shown in Fig. S5 exhibits a sharp increase at approximately 1656.1 K, indicating the onset of melting. Reported experimental melting temperatures for related CoCrFeNi‑based HEAs without Al generally fall in the range 1550-1700 K, while elemental Al melts at 933 K [1-4]. The simulated melting point therefore lies within the expected range based on known experimental data for constituent phases and similar alloy systems.

The quantitative consistency between the experimental measurements and the EAM based predictions, including lattice constants, cohesive energy trends, and melting temperature, demonstrates that the Co-Ni-Cr-Fe-Al-Cu EAM potential employed in the present work is reliable for modeling thermodynamic and structural properties of the investigated EHEA system.

**Supplementary Figures and Tables**


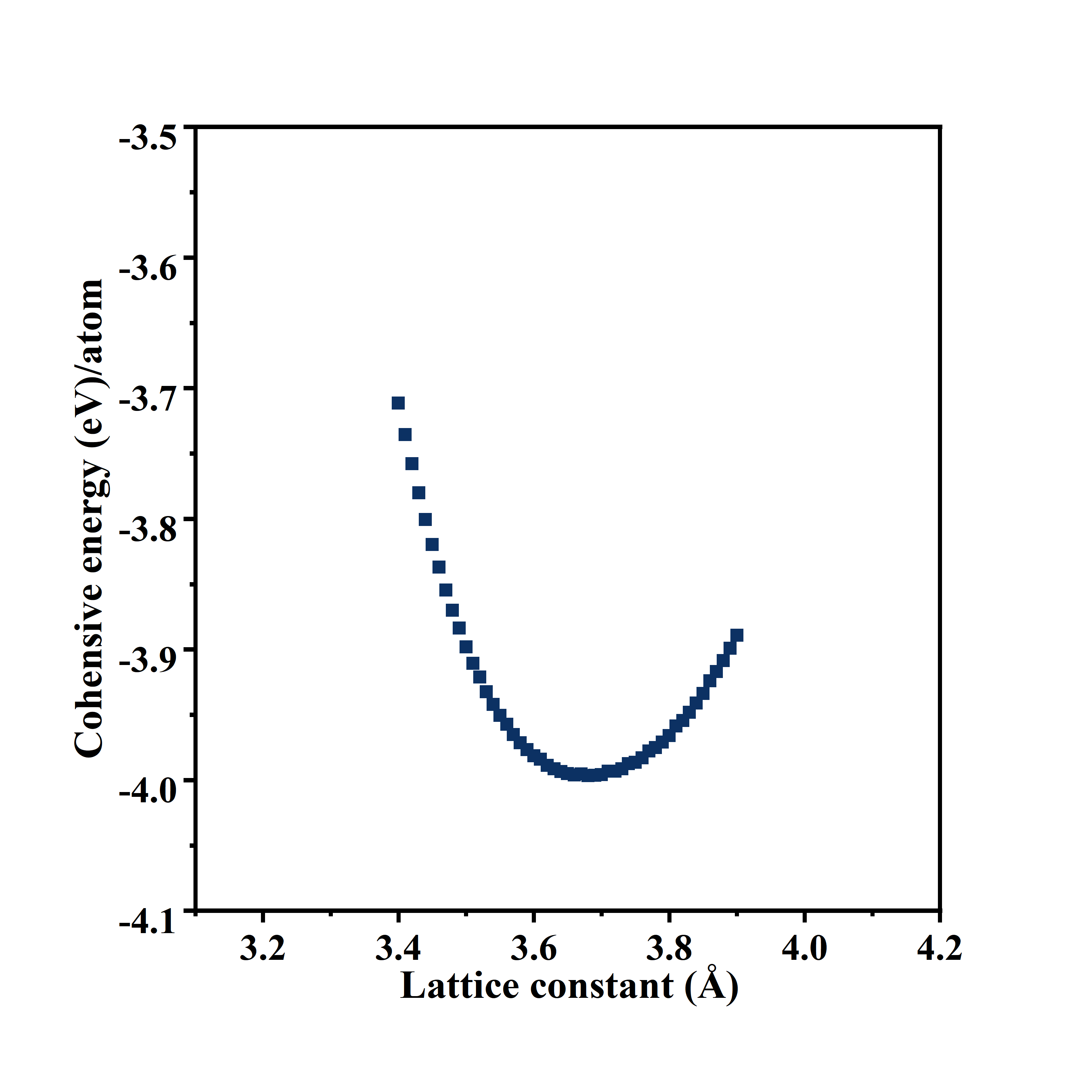

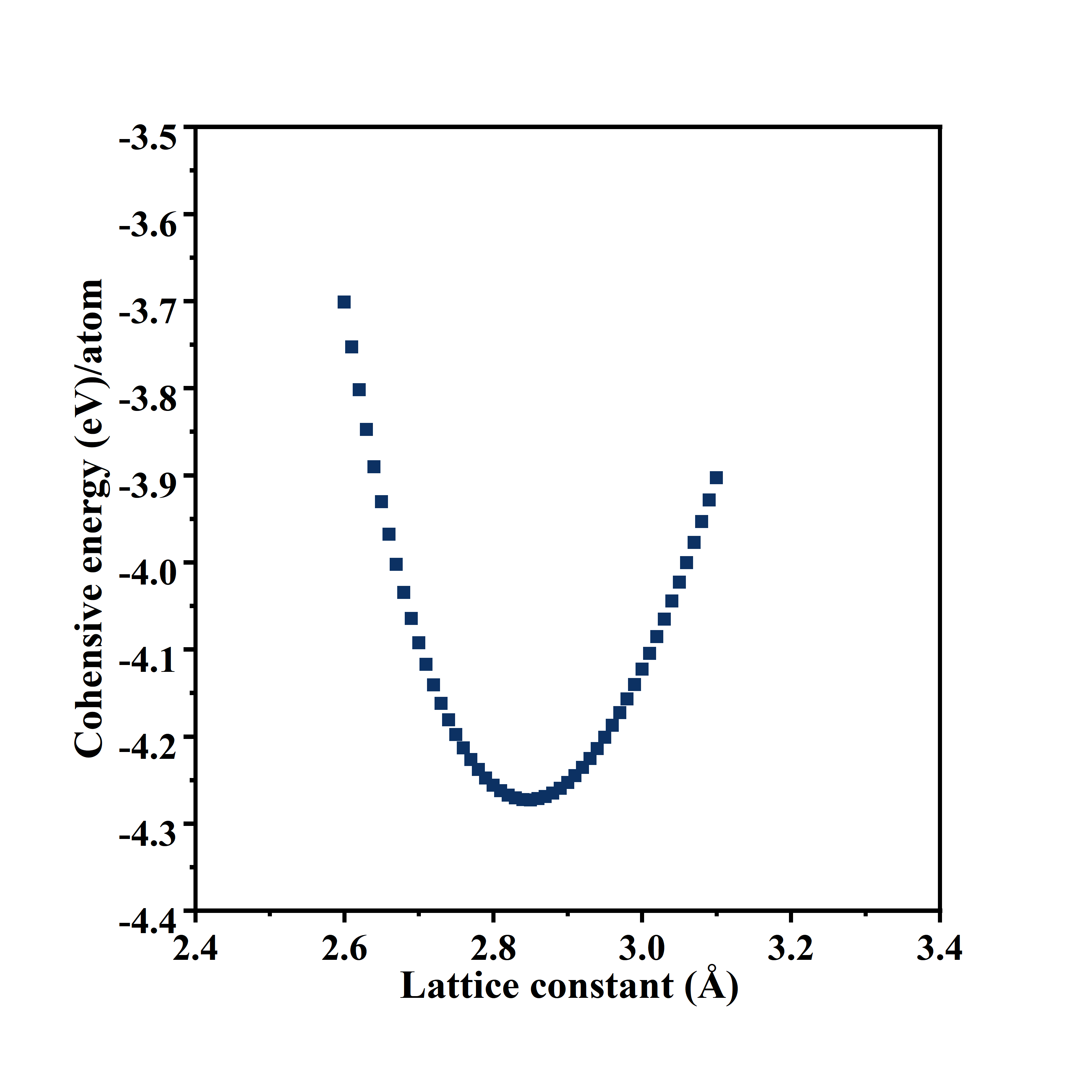

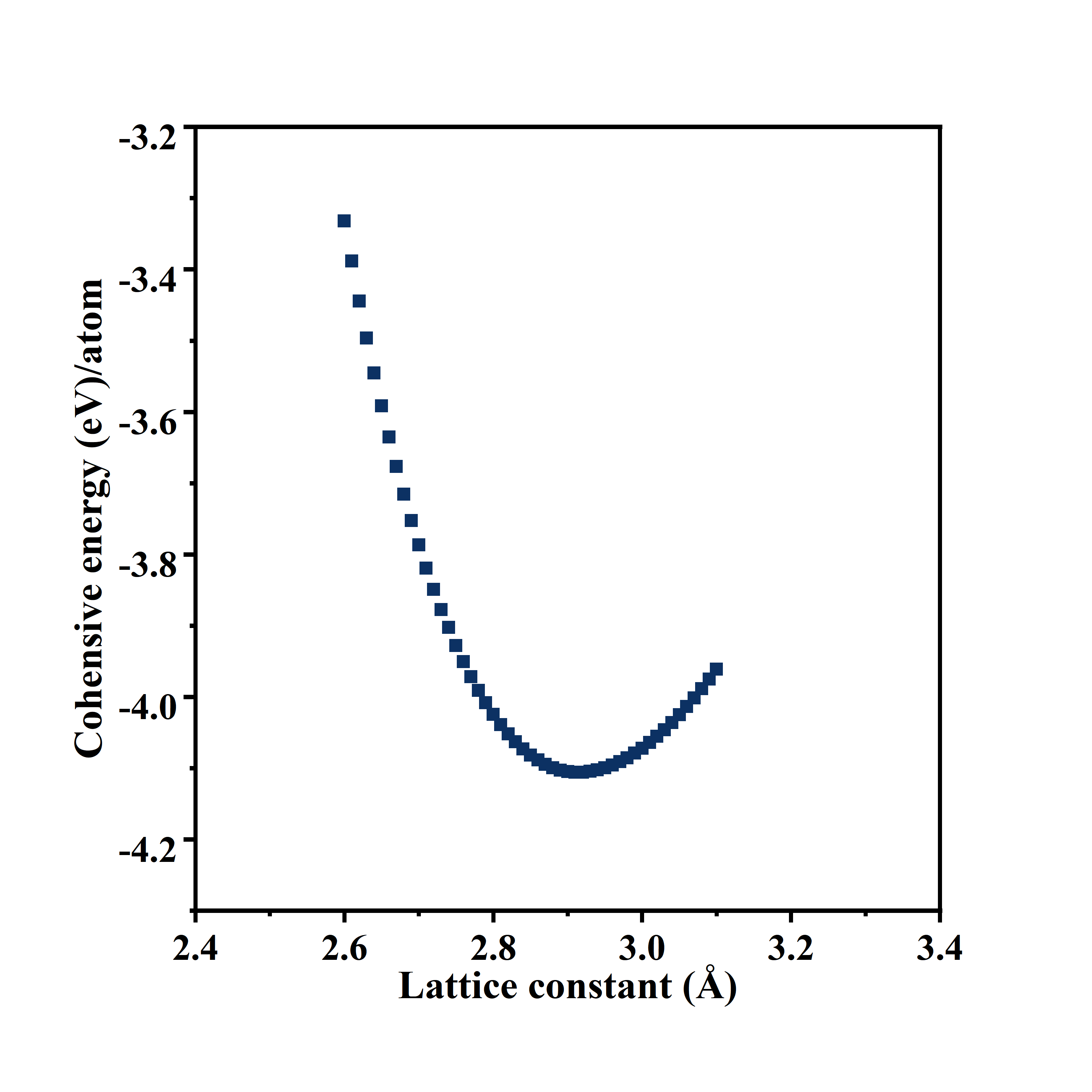


**BCC-FeCr**

**B2-NiAl**

**L1_2_**

Fig. S4 Cohesive energies of L1_2_, BCC-FeCr, B2-NiAl phase versus lattice constant.

Table S1 Comparison of cohesive energies of the B2 unit cell with MD simulations and DFT calculations.

| Compound | EAM (eV/atom) | DFT (eV/atom) | Deviation (%) |
| --- | --- | --- | --- |
| \| NiAl \| \| --- \| | -2.422 | -2.575 | 6.31 |
| \| FeAl \| \| --- \| | -2.482 | -2.582 | 4.05 |
| \| CoAl \| \| --- \| | -2.242 | -2.295 | 2.37 |
| \| CrAl \| \| --- \| | -2.294 | -2.332 | 1.66 |
| \| CuAl \| \| --- \| | -1.868 | -1.971 | 5.54 |


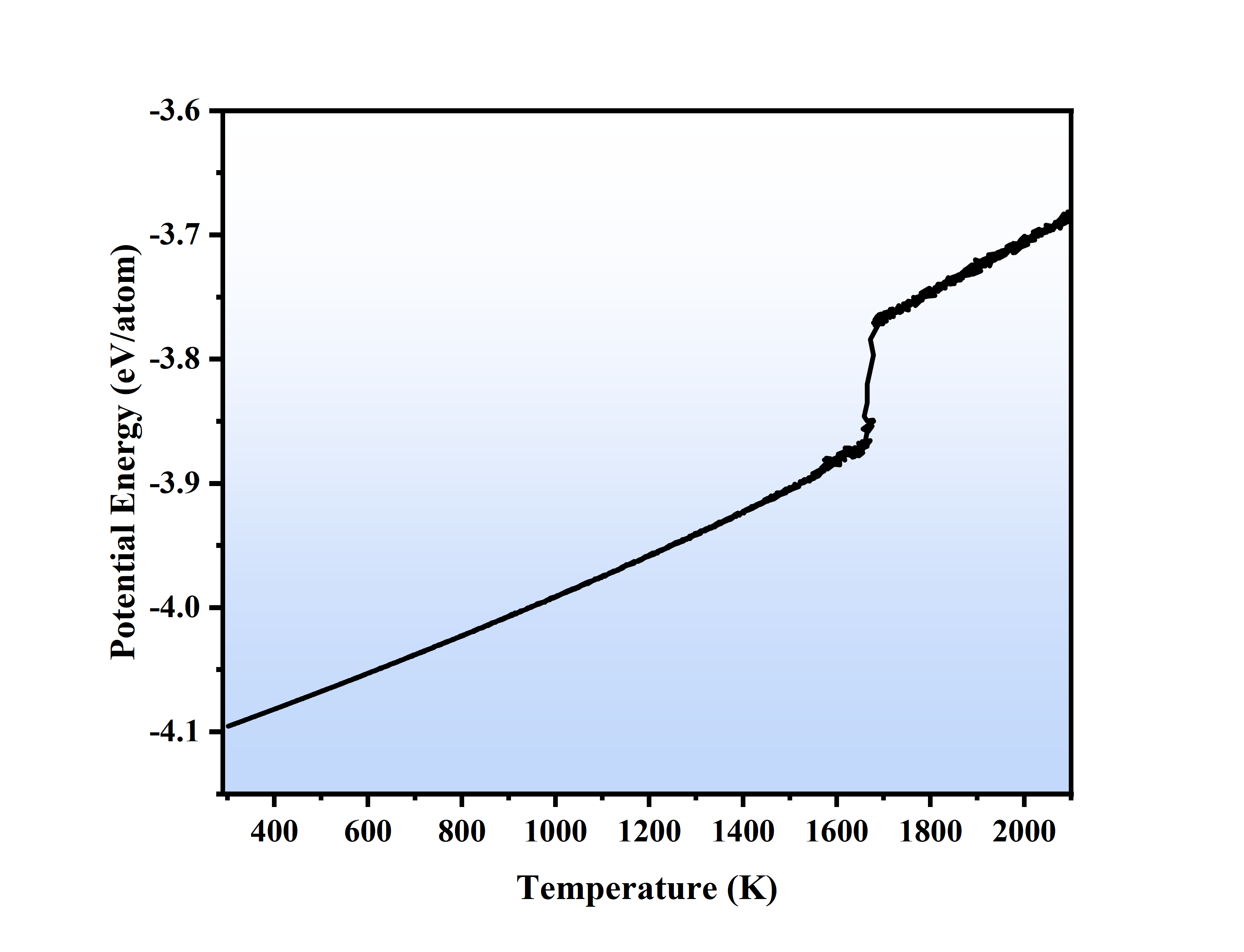


Fig. S5 Variation of potential energy with temperature for AlFeNiCoCrCu HEA

**Supplementary References**

[1] J. Dąbrowa, M. Zajusz, W. Kucza, G. Cieślak, K. Berent, T. Czeppe, T. Kulik, M. Danielewski, Demystifying the sluggish diffusion effect in high entropy alloys, J. Alloys Compd. 783 (2019) 193-207.

[2] A. Munitz, M. Kaufman, R. Abbaschian, Liquid phase separation in transition element high entropy alloys, Intermetallics 86 (2017) 59-72.

[3] C. Tong, Y. Chen, J. Yeh, S. Lin, S. Chen, T. Shun, C. Tsau, S. Chang, Microstructure characterization of Al_x_CoCrCuFeNi high-entropy alloy system with multiprincipal elements, Metall. Mater. Trans. A 36 (2005) 881-893.

[4] G.K. Sigworth, R.J. Donahue, The metallurgy of aluminum alloys for structural high-pressure die castings, Int. J. Metalcast. 15 (2021) 1031-1046.
